# Neutralization and ADCC reveal divergent spike-subdomain targeting across SARS-CoV-2 vaccine platforms in an African cohort

**DOI:** 10.1016/j.isci.2025.114351

**Published:** 2025-12-05

**Authors:** Gerald Kevin Oluka, Joseph Ssebwana Katende, Laban Kato, Violet Ankunda, Jackson Sembera, Peter Ejou, Geoffrey Odoch, Angella Namuyanja, Pontiano Kaleebu, Jennifer Serwanga

**Affiliations:** 1 Viral Pathogens Theme, Medical Research Council, Uganda Virus Research Institute and London School of Hygiene & Tropical Medicine (MRC/UVRI & LSHTM) - Uganda Research Unit, Entebbe, Uganda; 2 Department of Immunology, Uganda Virus Research Institute, Entebbe, Uganda; 3 London School of Hygiene and Tropical Medicine, Keppel Street, London WC1E 7HT, UK

**Keywords:** immunology, public health

## Abstract

Evaluating demographically diverse antibody response dynamics remains relevant to developing better vaccines against SARS-CoV-2 and related coronaviruses. Here, in 80 Ugandan study participants vaccinated with BNT162b2, CoronaVac or ChAdOx1-S, we investigated longitudinal neutralization and antibody-dependent cellular cytotoxicity (ADCC) functions, correlating these with immunoglobulin G (IgG) binding dynamics to spike subdomains, receptor-binding domain, N-terminal domain, and S2, across variants. Neutralizing and ADCC responses differed by vaccine type and IgG subdomain binding profiles. S2-IgG binding and ADCC effector function dominated the immune response to CoronaVac vaccination, while BNT162b2 induced the most potent neutralizing antibodies. Overall, we reveal intra-population diversity in antibody binding, neutralization and ADCC among vaccinated individuals, many of whom exhibited elevated pre-vaccination S2-IgG, despite presumed absence of prior infection. There was confounding by vaccination scheduling amidst ongoing waves of infection. These data reveal distinct immunogenetic patterns in a sub-Saharan African population that could inform regionally tailored vaccine strategies and global pan-coronavirus vaccine development.

## Highlights

• BNT162b2 elicited the broadest and strongest neutralizing antibody responses
• CoronaVac induced S2-biased IgG and robust ADCC despite modest neutralization
• High baseline S2-IgG revealed cross-reactivity in presumed infection-naive adults
• Neutralization and ADCC mapped to distinct spike subdomains across vaccines

## Graphical abstract

**Figure:**
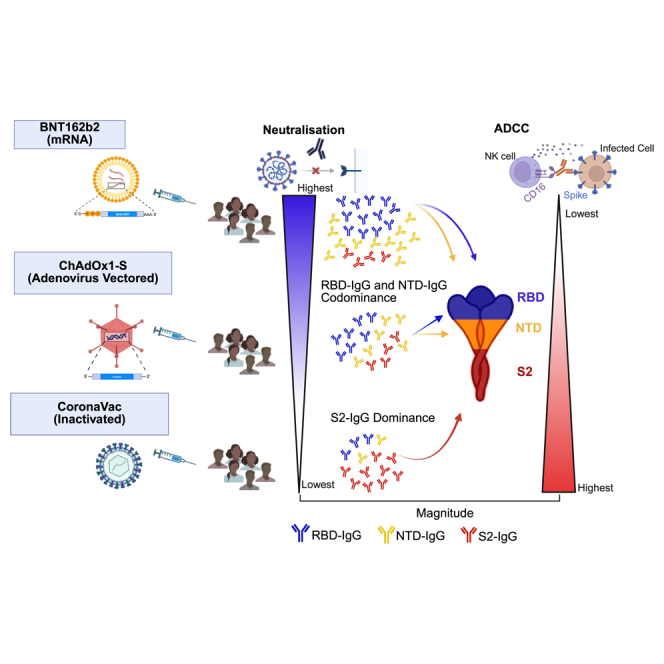

## Introduction

During the coronavirus disease 2019 (COVID-19) pandemic, several vaccine platforms were deployed globally, with many later updated to target emerging immune-evasive SARS-CoV-2 variants such as BA.4/5 and XBB.1.5.^1^^,^^2^ However, access to these updated bivalent formulations remained inequitable. In much of the global South, including Sub-Saharan Africa, populations received only the primary series of monovalent vaccines, such as the mRNA-based (BNT162b2 and mRNA-1273), inactivated (CoronaVac, BBV152, and BBIBP-CorV), or viral-vectored (Ad26.CoV2.S, ChAdOx1-S, and Ad5-nCov).^3^^,^^4^^,^^5^ Although early vaccine-induced neutralizing responses were detectable, the emergence of the B.1.1.529 (Omicron) variant substantially diminished neutralization sensitivity across most platforms.^6^^,^^7^ While some vaccine-induced responses persisted, emerging sub-lineages with greater antigenic divergence, such as XBB.1.5 and BQ.1.1, further diminished the neutralization sensitivity, rendering many initial vaccine-elicited responses largely ineffective.^8^^,^^9^^,^^10^ Despite global efforts to characterize vaccine-induced immunity, critical gaps persist in our understanding of the kinetics, durability and breadth of antibody responses across platforms, particularly non-neutralizing effector functions such as antibody-dependent cellular cytotoxicity (ADCC), and especially in underrepresented populations.^11^ Here, we present longitudinal immune profiling in an a Ugandan cohort,^3^^,^^4^^,^^5^ vaccinated with two doses of BNT162b2 (Pfizer-BioNTech), CoronaVac (Sinovac), or ChAdOx1-S (Oxford-AstraZeneca), capturing the temporal dynamics of spike-specific binding, neutralizing and ADCC antibody responses, before and after vaccination. These data reveal novel insights into the functional landscape of vaccine-induced immunity in a previously uncharacterized demographic.

The most potent and broadly neutralizing antibodies elicited by SARS-CoV-2 infection and vaccination predominantly target the spike (S) glycoprotein, which is the principal immunogen in current vaccine platforms.^12^^,^^13^ Despite its immunodominance, the spike protein is highly mutable, and substitutions within key neutralizing epitopes, particularly the receptor-binding domain (RBD) and N-terminal domain (NTD), have substantially diminished the neutralization breadth of many elicited antibodies.^14^^,^^15^^,^^16^ These antigenic shifts have driven global efforts to generate antibodies and immunogens with cross-variant breadth, aiming for pan-coronavirus protection.^12^^,^^17^ While additional booster doses transiently improve the elicited humoral immunity and partly restore neutralizing activity against immune evasive variants,^18^ the rapid emerging novel lineages continue to erode the efficacy of vaccine-induced neutralizing responses. Compounding this challenge is antigenic imprinting, where memory B-cell responses are preferentially recalled toward ancestral epitopes,^19^^,^^20^ even after exposure to updated antigens. As a result, reformulated mRNA vaccines such as BNT162b2 and mRNA-1273, incorporating Omicron-specific sequences, have required multiple booster doses to improve breadth of neutralizing antibodies.^1^^,^^21^

To advance the rational design of broadly protective vaccines, it is essential to characterize antibody responses across diverse demographic backgrounds. Such efforts are key to clarifying immunological mechanisms of protection against SARS-CoV-2 and related coronaviruses, particularly those targeting conserved antigenic sites within the spike, that could inform pan-coronavirus vaccine strategies.^17^ Recent studies have highlighted the emergence of S2-specific IgG antibodies with dual functionality, capable of mediating both neutralization and ADCC functions.^22^^,^^23^^,^^24^^,^^25^ Monoclonal antibodies with broad cross-reactivity and pan-coronavirus neutralizing activity have been isolated, targeting highly conserved regions of the spike protein, such as the NTD, the S2 stem helix, and the fusion peptide.^12^^,^^26^ These findings underscore the immunological significance of conserved epitopes as promising targets for next-generation vaccine design.

Importantly, accumulating evidence^27^^,^^28^^,^^29^^,^^30^ suggests that a substantial proportion of vaccine-induced antibodies are non-neutralizing, mediating protection primarily through Fc-dependent effector functions such as ADCC. These non-neutralizing antibodies often target conserved non-RBD epitopes within the S2 and NTD subunits,^31^^,^^32^ although the *in vivo* mechanisms underpinning their protective functions in humans remain incompletely understood.^11^ Multiple independent studies have reported robust ADCC activity following SARS-CoV-2 vaccination, directed against several variants of concern (VOCs).^33^^,^^34^^,^^35^ However, variability in response magnitudes and profiles highlight potential inter-population differences in Fc effector function. One study notably observed attenuated vaccine-induced ADCC compared to natural infection,^34^ implicating population-specific genetic, environmental, or immunological factors in modulating Fc-mediated immunity. Population-specific heterogeneity^13^^,^^36^ in the magnitude and quality of vaccine-induced antibody, across platforms and dosing regimens, has highlighted the need to optimize vaccine design for universal and durable protection.

**Figure 1:**
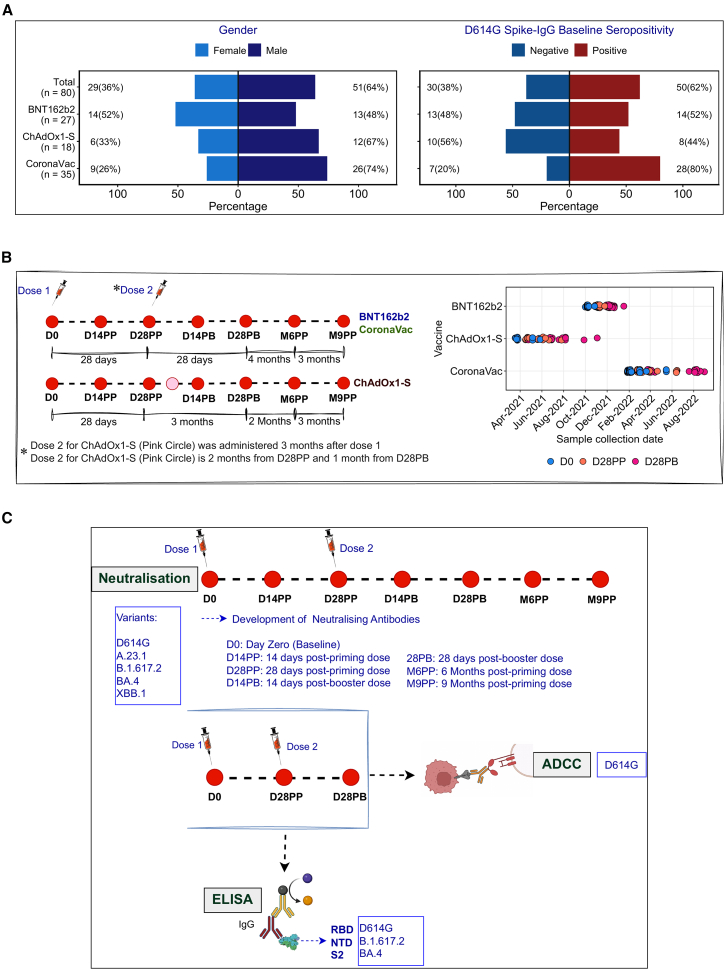
Summary of participant demographics and study design (A) Gender distribution and baseline spike-IgG serostatus categories of the study participants. (B) Vaccination schedules, longitudinal blood collection and plasma processing time points, and variation in timelines of sample collection for the three vaccines with reference to the periods of predominant national waves of infection by different variants.^37^^,^^38^ (C) Summary of the study design, highlighting time points used in neutralization, ADCC, and ELISA assays.

To address this, we evaluated the functional landscape of vaccine-induced antibodies in a uniquely stratified Ugandan cohort, all immunized with the ancestral spike-based vaccines, delivered through three distinct platforms: mRNA (BNT162b2), inactivated (CoronaVac), or adenoviral vector (ChAdOx1/nCoV-19). We longitudinally analyzed plasma collected over 9 months post-vaccination, delineating the magnitude, breadth, and interrelationships of antibody functions, including specificity to spike subdomains: RBD, NTD, and S2 (Figure 1). Our findings reveal striking platform-specific differences in functional immune profiles. Consistent with prior studies,^13^^,^^39^ BNT162b2 elicited the most potent and broadly neutralizing antibodies across all spike sub-domains. A key novel finding of this study is the strong ADCC activity elicited by CoronaVac, surpassing that of other platforms despite its relatively modest neutralization capacity. Notably, elevated S2-specific IgG were observed, implicating this conserved spike domain in cross-variant immunity and durable protection. These data underscore the value of population-specific immune profiling in informing next-generation vaccine design.

## Results

### Elevated baseline S2-IgG suggests preexisting cross-reactivity prior to vaccination

**Figure 2:**
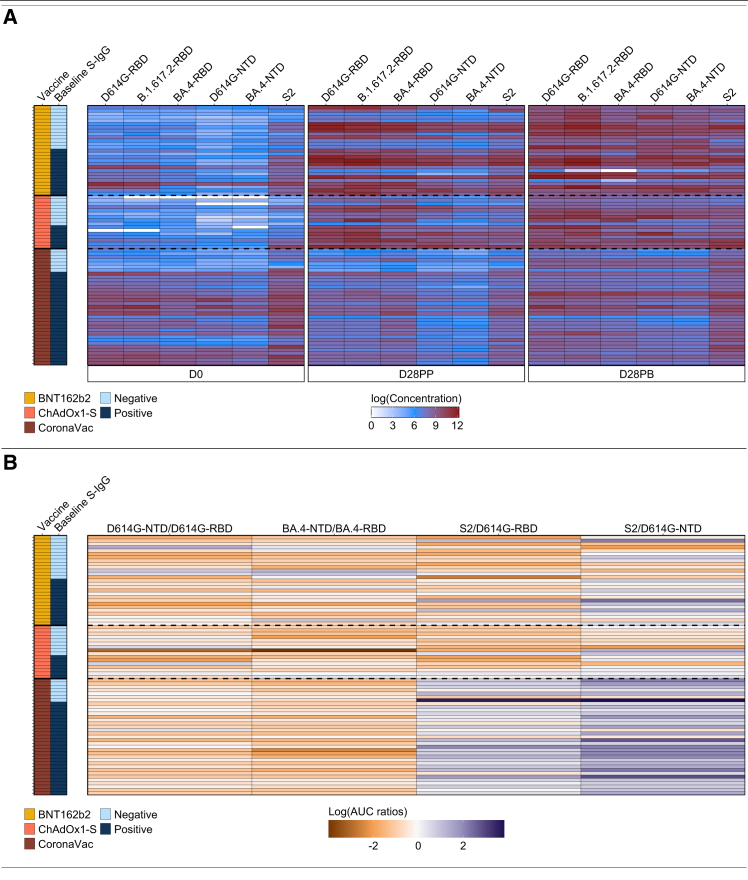
Participant-specific relative distribution of variant RBD, NTD, S2-IgG responses, and relative proportions of responses to antigens (A) This heatmap shows the relative distribution of IgG binding concentrations targeting RBD, NTD, and S2 subdomains across variant antigens at D0, D28PP, and D28PB for each participant. Color intensity ranges from white (lowest concentration) to red (highest concentration), illustrate inter- and intra-individual variation in magnitude and antigenic breadth over time. (B) Time-resolved ratios of binding concentrations between specified antigenic pairs, derived from AUC are shown for each participant as horizontal color gradients. Ratios <1 (decreased binding) are indicated by white to dark-orange gradients. Ratios >1 (increased binding) are indicated by white to dark-blue gradients. Ratios = 1 are denoted by white.

To assess vaccine-induced humoral responses across different platforms, we quantified plasma IgG binding antibodies targeting the RBD, NTD, and S2 subunits of the SARS-CoV-2 spike protein in 80 participants vaccinated with one of three vaccine platforms: the mRNA-based BNT162b2 (*n* = 27), adenovirus-vectored ChAdOx1-S (*n* = 18), or inactivated CoronaVac (*n* = 35). Antibody responses were assessed longitudinally at day 0 (D0, baseline), day 28 post-prime (D28PP), and day 28 post-boost (D28PB) (Figure 1). Binding was measured as optical density (OD; Figure S1) and corresponding antibody concentrations (ng/mL) (Figures 2 and S2). Notably, baseline S2-IgG levels were unexpectedly elevated across all vaccine platforms prior to vaccination. To distinguish vaccine-induced responses from those due to prior SARS-CoV-2 exposure,^40^^,^^41^ we stratified participants by baseline full-length D614G-S-IgG levels into seronegative (S-IgG−) and seropositive (S-IgG+) individuals (Figure 1A) based on previously established criteria.^42^ Corresponding N-IgG responses were also assessed (Figure S1) to differentiate prior or active exposure from true seronegativity.

At baseline, S2-IgG levels were consistently elevated in both baseline S-IgG− and S-IgG+ individuals across all three vaccine platforms (Figures 2 and S1). Among most infection-naïve individuals, baseline S2-IgG median OD values exceeded seropositivity thresholds, revealing potential prior exposure to antigenically related endemic human coronaviruses.^43^ Baseline IgG responses to RBD and NTD subdomains were negligible in infection-naïve individuals across all three vaccine platforms (Figure S1A). However, a subset of baseline S-IgG-participants across all vaccine platforms had elevated baseline RBD-IgG responses to D614G, B.1.617.2, and BA.4 variants, despite lacking full length spike seropositivity, like those observed for S2-IgG.

### Vaccine platform, dosing interval, and baseline serostatus shaped differential IgG binding dynamics to variant RBD, NTD, and S2 subdomains

Among BNT162b2 and ChAdOx1-S recipients, the most pronounced fold-increase post-priming occurred in RBD-IgG responses (Figure S3). In contrast, CoronaVac responses were still dominated by elevated S2-IgG (Figures 2 and S1A), with minimal early induction of RBD- and NTD-specific IgG. Following administration of the CoronaVac booster, infection-naïve individuals exhibited significantly higher IgG responses to RBD, NTD, and S2 by 28 days post-boost, compared to those with detectable IgG at baseline (Figures S2 and S3). Importantly, while the priming dose of CoronaVac-induced limited responses in infection-naïve participants, boosting elicited substantial increases in IgG binding titers across variant RBDs, NTDs, and S2 domains in the S-IgG– group (Figure S1).

### Spike RBD-, NTD-, and S2-specific IgG landscapes revealed intrapopulation diversity, and S2-biased targeting induced by CoronaVac

Evaluation of domain-specific antibody binding patterns elicited by the different vaccine platforms, stratified by vaccine type and baseline S-IgG serostatus, revealed intrapopulation heterogeneity, with divergent trajectories, even among individuals receiving the same vaccine and sharing similar baseline S-IgG serostatus. Antibody responses evolved non-uniformly, with peak binding concentrations occurring at differing time points, showing individual differences (Figure 2A).

To quantify and compare these sub-domain-specific IgG dynamics, we computed the time-resolved area under curve (AUC) values for each antigen-specific IgG trajectory using a log-linear trapezoidal method. AUCs were generated from vaccine-induced IgG concentrations against five antigens D614G-RBD, D614G-NTD, BA.4-RBD, BA.4-NTD, and S2 (D614G-S2), measured at D0, D28PP, and D28PB. To dissect the relative distribution of IgG responses across spike subdomains, we calculated pairwise AUC ratios between antigenically related regions: D614G-NTD: D614G-RBD, BA.4-NTD: BA.4-RBD, S2: D614G-RBD, and S2: D614G-NTD (Figure 2B). A dominant pattern emerged in most participants, characterized by higher RBD-IgG than NTD-IgG responses, and higher NTD-IgG than S2-IgG. This canonical pattern^27^ of RBD > NTD > S2 IgG (orange trajectories; Figure 2B) was broadly conserved across the cohort. In contrast, a distinct subset, exhibited non-canonical profiles (blue trajectories) characterized by disproportionately elevated NTD-IgG or S2-IgG concentrations relative to RBD-IgG. CoronaVac was found to elicit a predominant S2-IgG response (Figure 2B) in both baseline S-IgG− and S-IgG+ participants.

### Pre-vaccination RBD- and S2-directed IgG confers cross-variant neutralization in some spike-IgG-individuals, revealing a hidden layer of baseline immunity

To assess the breadth and evolution of neutralizing antibody responses against SARS-CoV-2, we performed spike-pseudotyped lentiviral neutralization assays to quantify neutralizing antibody titers (NT_50_) against five epidemiologically and antigenically distinct variants: D614G (ancestral wild-type), A.23.1 (Ugandan lineage),^20^^,^^37^ B.1.617.2 (Delta), BA.4 (Omicron subvariant), and XBB.1 (highly mutated Omicron lineage). Neutralization kinetics were evaluated at seven longitudinal time points: baseline (D0), 14- and 28-day post-primary dose (D14PP and D28PP), 14- and 28-day post-booster dose (D14PB and D28PB), then 6- and 9-month post-priming (M6PP and M9PP) (Figures 1B and 1C). To account for the impact of preexisting immunity, participants were stratified by baseline spike-specific IgG serostatus into seronegative (S-IgG−) and seropositive (S-IgG+) groups.

**Figure 3:**
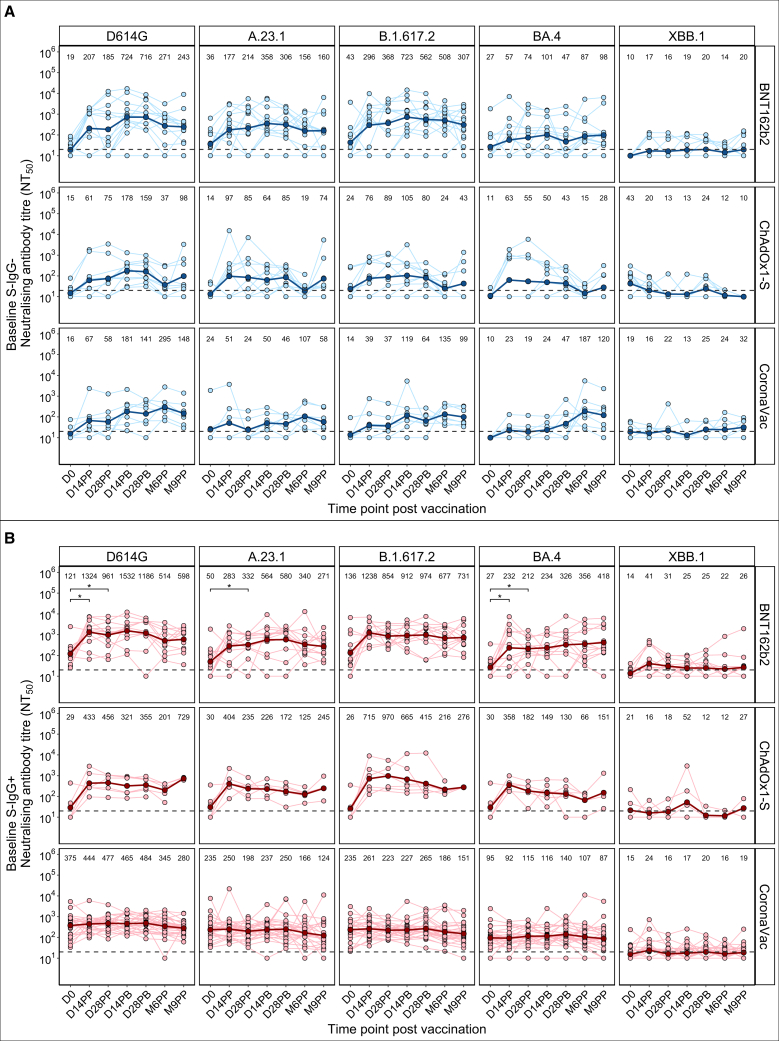
Longitudinal profiles of neutralizing antibody responses Neutralizing antibody titers at 50% inhibition (NT_50_) are shown over time for baseline S-IgG− (infection naive) participants (A) and baseline S-IgG+ (previously infected) participants (B) with each participant’s data points linked by thin blue (A) or red (B) lines. The dashed horizontal line indicates the assay’s lower limit of detection (NT_50_ = 20); values below this threshold were assigned a nominal NT_50_ value of 10, for analysis. Geometric mean titers (GMT) at each time point are displayed above each plot, with thick blue (A) or red (B) horizontal lines show GMT trends across time points. Statistical comparisons between GMTs at different time points were performed using pairwise Mann-Whitney U-tests with Benjamini-Hochberg correction for multiple testing. Statistical significance was set at *p* ≤ 0.05, denoted here as ∗(*p* ≤ 0.05).

After baseline neutralisation in S-IgG- individuals (*n* = 30) was undetectable or low across all variants, with geometric mean titers (GMTs) ranging from ≤20 to 43, near or below the assay’s lower limit of detection (NT_50_ = 20) (Figures 3A and S4A). Remarkably, 86.7% (26/30) of these presumed infection naive individuals exhibited measurable neutralization activity against at least one variant, with NT_50_ values ranging from 21.2 to 3758.9 (median = 74.9; Interquartile range (IQR) = 33.0–114.8), indicating potential presence of preexisting cross-neutralizing antibodies^44^ despite undetectable full spike IgG. To probe the source of this baseline neutralizing capacity, pre-defined OD thresholds OD ≥ 0.432 for S2-IgG and OD ≥ 0.178 for both RBD- and NTD-specific IgG (Figure S1),^42^ were applied. Samples with baseline neutralization (*n* = 50) demonstrated subdomain-specific IgG seropositivity, targeting B.1.617.2-RBD (*n* = 30), D614G-RBD (*n* = 28), S2 (*n* = 26), BA.4-RBD (*n* = 10), D614G-NTD (*n* = 5), and BA.4-NTD (*n* = 2), revealing existence of modest preexisting levels of cross-reactive, epitope-specific neutralizing antibodies, primarily directed against RBD and S2.

The mRNA vaccine, BNT162b2, elicited the broadest and most potent neutralizing antibodies, with the XBB.1 variant markedly evading neutralization.

Following the primary dose, all three vaccine platforms elicited substantial increases in neutralizing antibody titers (NT_50_) by day D14PP, with robust responses observed against the ancestral D614G strain and key variants A.23.1, B.1.617.2, and BA.4 (Figures 3 and S4). Among infection-naïve participants (Figure 3A), the mRNA-based BNT162b2 vaccine elicited the most pronounced fold increases in geometric mean titers (GMTs) with 10.9-, 4.8-, and 6.8-fold rises against D614G, A.23.1, and B.1.617.2, respectively (Figure S5A). In contrast, previously infected participants (Figure 3B) showed improved neutralization only with BNT162b2 and ChAdOx1-S, while CoronaVac yielded no substantial post-vaccination rise, maintaining moderate titers throughout follow-up (Figures 3B and S5B). Neutralization against the XBB.1 remained minimal or undetectable across all vaccine platforms and serostatus groups.

Neutralizing antibody titers peaked within 2 weeks post-booster, with the highest NT_50_ observed against D614G, followed by B.1.617.2, A.23.1, and BA.4 (Figure S4). Despite this peak, neutralization of XBB.1 remained negligible, even at peak post-booster time points, highlighting its extensive immune escape. Prior SARS-CoV-2 exposure conferred higher NT_50_ across all variants compared to infection naive individuals (Figure 3B), consistent with the superior breadth and magnitude afforded by hybrid immunity.^41^^,^^45^ In vaccine recipients with prior infection, titers plateaued post-booster, suggesting limited added benefit beyond infection-induced immunity. Nonetheless, BA.4 neutralization was higher in this group than in S-IgG-individuals. ChAdOx1-S recipients lacking prior exposure mounted only marginal post-booster NT_50_ increases (Figures 3A and S5A), likely due to the extended 12-week interval between the primary and booster doses (Figure 1), in contrast to the shorter 4-week schedules for BNT162b2 and CoronaVac.

Following booster administration, neutralizing antibody levels plateaued across all vaccine platforms, but a marked decline was observed by 6 months post-priming (M6PP), particularly in BNT162b2 and ChAdOx1-S recipients (Figure 3). Waning was most pronounced in the ChAdOx1-S group, which exhibited the lowest final GMTs across all variants. Notably, a sharp resurgence in neutralizing titers between M6PP and M9PP, especially against D614G, A.23.1, B.1.617.2, and BA.4, suggested breakthrough infections in ChAdOx1-S recipients during this interval^38^ (Figure 3A).

To assess neutralization quality, NT_50_ titers were stratified into five categories: undetectable (<20), low (20–200), medium (200–500), high (500–2,000) and potent (>2,000). The proportions (%) of participants in each category were then evaluated over time (Figure S6A). Consistent with quantitative titers, prior SARS-CoV-2 exposure significantly improved both breadth and potency of neutralization across platforms, with baseline S-IgG+ individuals attaining a higher proportion of participants with potent responses, even against the immune-evasive Omicron BA.4 variant (Figure S6B). Booster doses further improved the neutralization quality, as reflected by increased frequencies of participants achieving high and potent titers across multiple variants (Figures S6 and S7), thus supporting booster-based strategies. Among the tested variants, D614G and B.1.617.2 were the most effectively neutralised, with the highest proportions in the medium to potent range. A.23.1 and BA.4 elicited intermediate responses, while the XBB.1 variant was uniformly poorly neutralized, with titers largely undetectable or low across all vaccine platforms and serostatus groups (Figure S7). In line with binding profiles, neutralizing responses remained highly heterogeneous, reflecting substantial inter-individual variation in the magnitude and breadth of vaccine-induced immunity (Figure S7).

### Correlations between NT_50_ and IgG concentrations revealed differential distribution of neutralizing antibody responses to RBD, NTD, and S2 subdomains across vaccine types

To delineate the relationship between vaccine-elicited binding antibodies and neutralization, we performed Spearman’s rank correlation analyses between NT_50_ values and IgG concentrations targeting RBD, NTD, and S2 subunit subdomains. There were strong statistically significant positive correlations between NT_50_ titers and D614G spike-IgG concentrations, but not with nucleoprotein-IgG (Figure S8A), suggesting spike-centric antibody responses and limited influence from re-infections and breakthrough infections during follow-up (Figure S8B).

Subdomain analyses revealed a consistent hierarchy: NT_50_ values correlated most strongly with RBD-IgG and NTD, then S2 (Figure S9), aligning with established codominance of RBD and NTD in neutralization.^27^^,^^46^ However, BNT162b2 recipients also exhibited significant correlations between NT_50_ values and S2-IgG concentrations. Our previous analysis of IgG binding antibody distributions across the RBD, NTD, and S2 domains (Figure 2B) demonstrated that CoronaVac predominantly induced S2-focused IgG responses. However, correlation analyses showed minimal association with neutralizing titers, suggesting mediation of other effector functions.

### Inactivated CoronaVac vaccination and prior SARS-CoV-2 infection elicited stronger spike IgG-mediated ADCC than either ChAdOx1-S or BNT161b2

We longitudinally assessed spike IgG-mediated ADCC following BNT162b2, ChAdOx1-S, or CoronaVac vaccination. Responses were measured at baseline, 28 days post-prime dose and 28 days post-boost using a standardized ADCC assay^47^ in which HEK-293T/17 cells expressing the D614G spike served as targets and primary natural killer (NK) cells as effectors. Functional capacity was defined by NK cell degranulation, quantified by measuring CD107a surface expression, an established surrogate of cytotoxic potential.^33^^,^^48^

**Figure 4:**
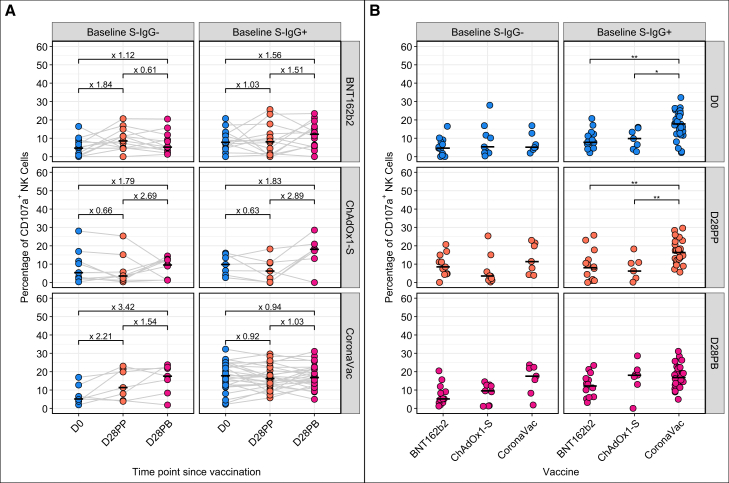
Longitudinal antibody-dependent cellular cytotoxicity profiles Longitudinal profiles of CD107a expression, a marker of degranulation and ADCC activity, are shown stratified by baseline S-IgG serostatus (A) and vaccine platform (B). Each data point represents an individual, with horizontal bars indicating group medians. Fold-changes in median %CD107a expression across time are annotated above each time point in (A). Statistical comparisons between paired time points were performed using the paired Wilcoxon test with Benjamini-Hochberg correction for multiple testing. Significance levels are indicated as ns (not significant, for *p* > 0.05, all not shown), ∗(*p* ≤ 0.05), and ∗∗(*p* < 0.01); for visual clarity, only statistically significant comparisons are shown (B).

In infection-naïve participants, baseline ADCC activity was uniformly low across all vaccine groups (Figure 4A). A small subset (*n* = 8, 0.1%) had elevated CD107a levels (≥10%), exceeding typical unvaccinated baselines,^33^^,^^49^ likely reflecting preexisting cross-reactive Fc-effector antibodies. Among individuals with baseline SARS-CoV-2 exposure (*n* = 32, 41%), baseline ADCC activity was markedly elevated (Figure 4B). These elevated responses coincided with heightened binding responses to the conserved S2 domain (median OD values: 1.207 [IQR; 1.038–1.353] compared to 0.603 [IQR; 0.206–0.789] in the infection-naïve individuals. Notably, 26 (65%) of the 40 participants with robust baseline ADCC (%CD107a ≥10%) were from the CoronaVac cohort, compared to only seven in each of the BNT162b2 and ChAdOx1-groups, highlighting cohort-specific differences in prior viral exposure.^38^

In infection-naïve participants, CoronaVac elicited the most pronounced ADCC induction following priming. Median CD107a expression increased 2.21-fold at D28PP, followed by a 1.55-fold rise post-boost, peaking at 17.53% (IQR; 12.0–22.10), a 3.42-fold elevation from baseline (Figure 4A). Among the baseline seropositive participants, CoronaVac sustained high ADCC levels post-vaccination (Figure 4B), aligning with our neutralization data (Figure 3B), and suggesting that preexisting immunity may potentiate ADCC up to a functional homeostatic ceiling.^50^ In contrast, there was no ADCC induction following the ChAdOx1-S priming dose, but was significantly increased post-boost; 2.69-fold in naive and 2.89-fold in seropositive individuals. Peak responses in previously exposed participants (median: 18.05%; IQR; 15.2–19.6) approximated those elicited by CoronaVac (Figure 4). BNT162b2, however, elicited consistently low ADCC across baseline serostatuses and at the different time points, suggesting a comparatively lower capacity of mRNA-elicited antibodies to engage NK cell-mediated cytotoxicity, despite robust neutralizing activity.

The longitudinal tracking of N-IgG fold changes (Figure S8B) suggests that the potent ADCC function and S2-IgG antibodies elicited by CoronaVac may contribute to protection despite weaker neutralization. Participants vaccinated with CoronaVac were not any more susceptible to the breakthrough infections (*n* = 8), compared to BNT162b2 (*n* = 11) or ChAdOx1-S (*n* = 8) which induced more potent neutralizing antibodies (Figures 3A and S8B).

### Polyfunctionality of CoronaVac-induced antibodies was highlighted by correlations of ADCC effector function with neutralizing and binding antibody response profiles

To assess the functional breadth of humoral immunity elicited by CoronaVac, we longitudinally profiled the evolution of ADCC alongside binding and neutralizing antibody responses. Using the log-linear trapezoidal method, we calculated area under the curve (AUC) values for each participant across baseline, 28 days post-prime and 28 days post-boost time points, capturing CD107a+ NK cell activation and NT_50_ titers against five SARS-CoV-2 variants. These data were integrated with spike-specific IgG binding AUCs (Figure S10), enabling comparison of functional antibody responses. Inter-participant heterogeneity was observed across IgG binding, ADCC, and neutralizing responses. Despite this, concordance between ADCC and neutralization activity was evident in several participants, with dominant polyfunctionality of CoronaVac-induced antibodies. Among the spike subdomains, S2-specific IgG AUCs had the strongest association with CD107a+ NK cell function, reinforcing the contribution of non-RBD targets in Fc-effector functions.^35^

## Discussion

In this study, we extended previous findings,^13^^,^^27^^,^^34^ by providing unique longitudinal insights into subdomain-specific spike IgG binding, neutralization, and Fc-effector functionality following vaccination in a Sub-Saharan population with prior exposure to endemic coronaviruses. Our investigation, which assessed responses to three different vaccine platforms, BNT162b2, ChAdOx1-S, and CoronaVac, offers important insight into how vaccine-induced humoral immunity unfolds in immunogenetically diverse populations, often underrepresented in global vaccine trials. The exclusive administration of wild-type spike-based vaccines in this setting, and the lack of access to variant-adapted boosters, presented an opportunity to examine the breadth, cross-reactivity, and Fc-effector functionality of vaccine-induced immunity in an African cohort.

We found a high prevalence of preexisting S2-IgG and in some cases RBD-IgG, likely reflecting previous exposure to endemic coronaviruses.^26^^,^^43^^,^^44^^,^^51^ Our observation of CoronaVac’s dominant S2-IgG profile, likely driven by high baseline titers, aligns with pre-clinical murine findings demonstrating that prior coronavirus exposure can imprint the immune system to preferentially boost conserved, subdominant regions such as S2.^22^ Interestingly, our data diverged from previous studies that indicated poor S2 induction after ChAdOx1-S vaccination,^52^ instead showing substantial S2-specific responses in this group. This discrepancy highlights the influence of population-specific immunogenetic landscape, possibly rooted in baseline memory B cell repertoires, specificity, and functional quality of vaccine-induced immunity.

Among the three vaccine platforms, BNT162b2 induced the highest magnitude of total spike-IgG, with broader subdomain-binding and superior neutralizing capability, which is consistent with existing literature.^13^^,^^36^^,^^39^ This immunogenic advantage likely reflects optimized spike-antigen delivery, the pre-fusion stabilized spike design in BNT162b2,^53^^,^^54^^,^^55^ and optimized spike antigen expression in ChAdOx1-S.^56^ Conversely, the lower antibody-response levels observed following CoronaVac vaccination may partly be the result of the alum-only adjuvant formulation, where novel AS03 or CpG-based adjuvants could meaningfully enhance antibody responses.^57^^,^^58^ While we observed substantial cross-neutralization against BA.4 in all vaccine platforms, XBB.1 escape was nearly complete, confirming its immunoevasive mutations and hybrid lineage.^2^ These data highlight the limitations of ancestral spike-based vaccines particularly in regions where access to updated boosters remained limited.^1^

All vaccines elicited measurable ADCC responses, with the most pronounced functions seen in CoronaVac recipients. This strongly correlated with elevated S2-IgG abundance and aligns with prior reports linking enhanced ADCC responses to natural infection,^34^ likely driven by the structural mimicry between the vaccine virions and live virus. Previous studies have shown that conserved epitopes within the S2 and NTD regions serve as dominant targets of Fc-mediated anti-viral effector function.^32^^,^^59^ In contrast, BNT162b2 showed modest post-vaccination increases in ADCC observed following immunization, despite robust spike-specific IgG responses, potentially due to saturation effects in individuals with preexisting ADCC-competent antibodies.^34^^,^^50^ Many individuals in this cohort likely had ADCC-competent antibodies at baseline, shaped by prior SARS-CoV-2 exposure or cross-reactivity from seasonal coronaviruses, resulting in limited augmentation post-immunization. This is reminiscent of prior observations in studies of influenza vaccination, where healthy, seronegative individuals exhibited robust baseline ADCC potential that was only marginally enhanced upon immunization.^60^^,^^61^ Nonetheless, our peak post-vaccination ADCC levels exceeded those reported in convalescent individuals,^49^^,^^62^ and matched or surpassed those elicited by other vaccine platforms,^33^^,^^35^^,^^49^ affirming the functional potency of vaccine-induced Fc responses in this African cohort.

While we suggest that potent ADCC function and S2-IgG binding by CoronaVac-elicited antibodies could have made up for lower neutralization potency, our findings are limited by smaller sample sizes of re-infection and breakthrough infections, as well as potential misclassification by reliance on N-IgG fold-change thresholds rather than rt-PCR and sequence verification. Better powered prospective studies would be necessary determine this conclusively by integrating functional assays such as ADCC with long-term breakthrough outcomes. Furthermore, some variations in antibody responses at baseline across vaccine platforms is attributable to confounding by the periods in which different vaccines were introduced into the population amidst ongoing waves of infection and re-infection, resulting in varied pre-vaccination histories.

Summarily, these findings underscore the importance of characterizing preexisting immunity, particularly cross-reactive Fc-effector memory, in vaccine evaluation frameworks. From a policy perspective, they advocate for integration of Fc-function profiling in immunogenicity assessments, especially in populations with baseline exposure and suggest the need to incorporate population-optimized strategies in vaccine design as well as refinement of vaccine platforms to improve neutralization potency and breadth as well as antibody effector functions.

### Limitations of the study

This study had some limitations that should be considered when interpreting our findings. First, the cohort comprised predominantly young adults, with a median age of 27 years, reflective of Uganda’s demographic structure, but not representative of older populations in which the magnitude and quality of responses might be differ. Furthermore, the cohort of participants was predominantly male and gender distribution across vaccine groups was not within the study’s controllable variables. Second, the three different vaccines were administered at varying periods of the SARS-CoV-2 infection-waves in the country^38^—ChAdOx1-S was introduced earliest in April 2021, shortly followed by BNT162b2. As such, about half of the recruited study participants were seronegative prior to vaccination. Conversely, CoronaVac was introduced much later after more than three national waves and peaks of infections, during which most study participants were likely to have been infected multiple times prior to receiving the first vaccine dose. This imposed variation in the proportions of baseline serostatuses across the three vaccines as well as observed baseline antibody levels. Second, ADCC functionality was assessed using a single spike variant (D614G), and thus the breadth of Fc-effector responses across contemporary variants, including XBB.1.5, remains unresolved. Moreover, we did not evaluate other Fc-dependent mechanisms such as phagocytosis and complement-mediated functions, as well as corresponding T cell-mediated functions which are key correlates of antiviral protection. Finally, follow-up mechanistic studies involving S2-specific IgG depletion assays, monoclonal antibody isolation and cross-coronavirus neutralization testing could provide a clearer understanding of the functional specificity of the antibody responses observed in this study.

## Resource availability

### Lead contact

Requests for further information and resources should be directed to and will be fulfilled by the lead contact, Jennifer Serwanga (jennifer.serwanga@mrcuganda.org).

### Materials availability

This study did not generate new unique reagents.

### Data and code availability

• All data reported in this paper will be shared by the lead contact upon request.
• This paper does not report original code.
• Any additional information required to reanalyze the data reported in this paper is available from the lead contact upon request.

## Acknowledgments

We would like to thank the participants in the Ugandan COVID-19 immunoprofiling cohort for providing the samples used in this study. We thank Prof. Katie Doores (King’s College London) for critical reading of the manuscript and providing antigen plasmids and cell lines, and Dr. Jerry C.H. Tam (King’s College London) and Dr. Dieter Mielke (10.13039/100006510Duke University) for technical advice on setting up the ADCC assays.

## Author contributions

G.K.O. and J. Ser conceptualized the study; P.E., G.O., and A.N. recruited study participants, collected, processed, and curated blood samples used in this study; G.K.O., J.S.K., L.K., and J. Sem performed laboratory experiments; V.A. and G.K.O. analyzed the data and created the figures; J. Ser and P.K. obtained funding and supervised the study; G.K.O. wrote the draft manuscript; J. Ser and P.K. revised and edited the manuscript. All authors read, subedited, and approved the manuscript.

## Declaration of interests

The authors declare no competing interests.

## STAR★Methods

### Key resources table

**Table:** 

REAGENT or RESOURCE	SOURCE	IDENTIFIER
**Antibodies**		

Monoclonal ANTI-FLAG® M2-FITC antibody	Sigma-Aldrich	Cat# F4049.2 MG; RRID: AB_439701
FITC Mouse Anti-Human CD107a	BD Biosciences	Cat# 555800; RRID: AB_396134
Pacific Blue™ Mouse Anti-Human CD16	BD Biosciences	Cat# 558122; RRID: AB_397042
PECy7 Mouse Anti-human CD56	BD Biosciences	Cat# 335809; RRID: AB_399984
Purified anti-human CD16 Antibody	BioLegend	Cat# 302002; RRID: AB_314202
CR3022 IgG1 Monoclonal antibody	Prof. David Veesler (USA)	–
Purified human IgG	Sigma-Aldrich	Cat# I2511; RRID: AB_1163604

**Bacterial and virus strains**		

JM109 competent cells	Promega	Cat #L2005
SARS-CoV-2 Pseudotyped viruses— D614G, A.23.1, B.1.617.2, BA.4, XBB.1 Variants	This paper	–

**Biological samples**		

Serum samples from BNT162b2, ChAdOx1-S and CoronaVac vaccinated human participants	Uganda Virus Research Institute	–
Primary NK Cells from a healthy donor	Uganda Virus Research Institute	–

**Chemicals, peptides, and recombinant proteins**		

LIVE/DEAD Fixable Aqua Dead Cell Stain Kit	Thermofisher Scientific	Cat# L34966
BD GolgiStop™ Protein Transport Inhibitor (Containing Monensin)	BD Biosciences	Cat# 554724
BD GolgiStop™ Protein Transport Inhibitor (Containing Brefeldin A)	BD Biosciences	Cat# 555029
Human IL-15 Research Grade	Milteny	Cat# 130-095-760
Human IL-2 Research Grade	Milteny	Cat# 130-097-743
PolyFect Transfection Reagent	Qiagen	Cat# 301107
Cell Activation Cocktail (without Brefeldin A)	Biolegend	Cat# 423302
Recombinant D614G spike S2 subunit protein	R&D Systems	Cat#10584-CV
PEI Max 40 000	Polysciences	Cat # 24765-1
Recombinant SARS-CoV-2 RBD and NTD	This paper	–
Phorbol 12-myristate 13-acetate and ionomycin (PMA/Ionomycin) cell activation cocktail	Biolegend	Cat# 423301
3,3′,5,5′-Tetra-methyl benzidine (TMB) substrate	Seracare	Cat# 5150-0021
PolyFect transfection reagent	Qiagen	Cat# 301105

**Critical commercial assays**		

NK Isolation Reagent Kit	Milteny	130-092-657
Bright-Glo luciferase kit	Promega	Cat#: E2610
Nickel-Nitrilotriacetic acid (Ni-NTA)	Qiagen	Cat# 30410

**Experimental models: Cell lines**		

FreeStyle™ 293F Cells	Thermofisher Scientific	Cat#: R79007
Human Embryonic Kidney (HEK)-293T/17 Cells	ATCC	ATCC® CRL-11268
HEK-293T/17-hACE-2	Prof. Katie Doores (UK)	–

**Recombinant DNA**		

pCAGGS-SARS-CoV-2 Spike D614G	Addgene^47^	Cat# 156420
SARS-CoV-2 RBD, NTD (pHLsec) for variant recombinant protein expression	Prof. Katie Doores (UK)	–
HIV-Gag/Pol p8.91	Prof. Katie Doores (UK)	–
Full length SARS-CoV Spike (pcDNA3.1+) for pseudotyped viruses	Nexelis- IQVIA Labs (Canada)	http://www.nexelis.com/
pHIV-Luciferase plasmid	Prof. Katie Doores (UK)	–
HIV-Gag/Pol p8.91	Prof. Katie Doores (UK)	–

**Software and algorithms**		

FlowJo	BD Biosciences	https://www.flowjo.com/
R	The R Foundation	https://www.r-project.org
R studio	R studio	https://www.rstudio.com/
Biotek GEN5 software	Agilent	–

**Other**		

FACS Melody	BDBiosciences	–
Victor ×3 multilabel reader	Perkin Elmer	–

### Experimental model and study participant details

#### Human participants

A total of 80 Ugandan participants were vaccinated with two doses of one of three SARS-CoV-2 vaccines; mRNA (BNT162b2), inactivated (CoronaVac), or viral-vectored (ChAdOx1-S), and followed longitudinally over 9 months, between April 2021 and August 2023.^3^^,^^4^^,^^5^ The three vaccines became available to the Ugandan population at different time frames; ChAdOx1-S was the earliest vaccine to be rolled out in April 2021 before the B.1.617.2 SARS-CoV-2 infection wave.^20^^,^^38^ This was shortly followed by the BNT162b2 during the course of the B.1.617.2 wave of infections. CoronaVac on the other hand, was introduced much later after more than three waves of infection, at which point most study participants had been infected prior to vaccination. Plasma samples were collected before the first vaccine dose (D0), 14 and 28 days after the prime dose (D14PP and D28PP), 14 and 28 days after the booster dose (D14PB and D28PB), then 6 and 9 months after the prime dose (M6PP and M9PP). Based on previously measured IgG responses to the full-length D614G spike protein at D0, 62% of the participants were classified as baseline-seropositive (S-IgG+) and presumed to have previously been infected. Participant demographics, vaccination schedule and study design are summarised in Table S1 and Figure 1. Before enrollment, written informed consent was obtained from all participants and study approval granted by Uganda Virus Research Institute’s Research and Ethics Committee (UVRI-REC Ref; GC/127/20/04/773), Makerere University School of Biomedical Sciences’ Research and Ethics Committee (SBS-REC, Ref: SBS-2024-571) and Uganda National Council for Science and Technology (HS997ES).

#### Bacteria strains and cell culture

All antigen plasmids were expanded by Maxipreps from transformations of competent bacterial cells. HEK-293F cells used in protein expression were maintained in FreeStyle expression media at 1 × 10^6^ cells/mL, with regular shaking at 37°C, 10% CO_2_ in a humidified incubator. HEK-293T/17 (ATCC CRL-11268), and HEK-293T/17-hACE-2 cells were cultured in Dulbecco’s modified Eagle’s medium (DMEM) supplemented with 10% fetal bovine serum (FBS), 1% penicillin–streptomycin and 0.4% puromycin at 37°C, 10% CO_2_ in a humidified incubator. Frozen stocks of cell lines were tested for mycoplasma contamination. Cell lines were not authenticated internally as they were all originally sourced from a non-profit company (ATCC). Primary natural killer (NK) cells from a healthy donor’s peripheral blood mononuclear cells (PBMC) were rested overnight in Roswell Park Memorial Institute (RPMI) 1640 complete medium supplemented with 10% fetal bovine serum (FBS), 1% Penicillin-Streptomycin, with stimulation by 10 ng/mL of Interleukin-15.

#### Peripheral blood processing and natural killer cell enrichment

Blood samples in citrate preserve were obtained from a healthy control donor, and peripheral blood mononuclear cells (PBMC) isolated by density-gradient centrifugation (Histopaque-1077), washed in Hanks’ Balanced Salt Solution (HBSS), and cryopreserved in Dimethyl sulfoxide (DMSO) with 10% FBS. Thawed cells were resuspended in supplemented complete medium (RPMI), and following an overnight rest at 2 × 10^6^ cells/mL, primary CD56^+^CD16^+^ NK cells were enriched by negative selection in magnetic activated cell sorting (MACS).

### Method details

#### Antigen expression and purification

HEK-293F Cells were transfected with 6xHis-tagged pHL-sec plasmids encoding stabilised ectodomains of SARS-CoV-2 variant (D614G, B.1.617.2 or BA.4) spike RBD or NTD, using polyethylenimine transfection reagent (PEI-Max, 1 mg/mL solution). Cell-culture supernatant was harvested after 6 days, clarified by centrifugation, and filtered through a 0.45 μm membrane. Recombinant spike protein was then purified by Nickel-Nitrilotriacetic acid (Ni-NTA) affinity chromatography, concentrated through 50 kDa molecular weight cut-off filters, buffer-exchanged into Phosphate Buffered Saline (PBS) and stored at −80°C till use.

#### RBD, NTD and S2-IgG enzyme-linked immunosorbent assay (ELISA)

As previously described,^42^ an in-house indirect ELISA was used to detect and quantify IgG antibodies targeting the RBD (D614G, B.1.617.2, BA.4), NTD (D614G, BA.4) and S2 (D614G)-subunits of the SARS-CoV-2 spike protein. Briefly, flat-bottomed medium-binding plates were coated with 3 μg/mL of antigen overnight at 4°C. The plates were washed with Phosphate Buffered Saline (PBS) containing 0.05% Tween 20 (PBS-T) and blocked with PBS-T containing 1% Bovine Serum Albumin (BSA) for 1 h at room temperature. Heat-inactivated plasma samples were added in duplicates at 1:100 and incubated for 2 h at room temperature, alongside predetermined positive and negative assay controls, as well as serial dilutions of standard purified human IgG at known concentrations. Following a wash step, plates were incubated with horseradish peroxidase-conjugated, goat anti-human IgG (HRP-IgG) for 1 h at room temperature, and after a final wash step, detected by 50 μL of 3,3′,5,5′-Tetra-methyl benzidine (TMB) substrate. After 3 min, the reaction was stopped by 50 μL of 1M Hydrochloric acid, and optical densities (OD) at 450 nm determined with a BioTek ELx808 microplate reader using BioTek GEN5 software. Net OD (OD450) values at 450 nm were obtained by subtracting blank-well OD values from sample wells, and antibody concentrations (ng/mL) obtained through extrapolation from a non-linear 4-parameter logistic (4-PL) standard curve of the serially diluted standard purified human IgG.

#### Production of SARS-CoV-2 pseudotyped-viruses

Replication-defective HIV-1 (lentiviral-based) proviruses containing the firefly luciferase (FLuc) reporter gene were pseudotyped with SARS-CoV-2 D614G, A.23.1, B.1.617.2, BA.4 or XBB.1 variant spike protein. T-75 culture flasks were seeded with 4.0 × 10^6^ HEK-293T/17 cells. After 24 h, culture media was refreshed and the cells co-transfected with 15 μg of pHIV-Luciferase plasmid, 10 μg of HIV-Gag/Pol p8.91 plasmid and 5 μg of variant spike protein plasmid using 100 μg of Polyethylenimine (PEI-Max) transfection reagent. Supernatant was collected after 72 h, clarified by centrifugation, and filtered through a 0.45 μm membrane, then stored at −80 °C ahead of the neutralisation assays. Pseudotyped-virus working titers were determined by titrations in 2-fold dilution series each with 10,000 HEK-293T ACE-2 cells, along with cell-only negative controls. Assay titers were normalised based on relative luminescence unit (RLU) outputs from the dilutions.

#### Pseudotyped-virus neutralisation assay

Longitudinal development of neutralising antibodies was measured by determining the inhibitory activity of antibodies against infection of HEK-293T/17 cells stably expressing the human ACE-2, by lentiviral-system pseudotyped variants of SARS-CoV-2; D614G, A.23.1, B.1.617.2, BA.4 and XBB.1. Pre-determined dilutions of pseudotyped viruses were incubated with heat-inactivated plasma samples in duplicates, at 3-fold serial dilutions ranging from 1:20 to 1: 43,740 for 1 h at 37°C in a humidified 5% CO_2_ incubator. HEK-293T-ACE-2 cells were then added at 10,000 cells per well and further incubated for 72 h. Promega 1× lysis buffer and Luciferase substrate were then added, to measure luciferase expression in the infected mammalian cells, using a PerkinElmer Victor ×3 luminometer. Neutralising antibody titers at 50% inhibition/neutralisation (NT_50_) were determined from relative luminescence units by fitting a non-linear regression curve.

To identify potential confounding by non-spike specific neutralising activity against the spike-pseudotyped HIV-1 proviruses in a general population where participants living with HIV would be on anti-retroviral (ARV) therapy, a cross-section of all participant samples were evaluated using a recombinant Env protein pseudotyped amphotropic murine leukemia virus (aMLV) in a TZM-bl (JC.53bl-13) cell neutralisation assay.^63^ Participants (*n* = 5) with NT_50_ ≥ 60, that is 3-fold above the assay’s lower limit of detection (NT_50_ = 20) were excluded from further analysis of neutralising antibody responses.

#### Antibody-dependent cellular cytotoxicity (ADCC)

The ADCC assay was adapted from Mielke et al.^47^ HEK-293T/17 cells (8.0 × 10^6^ cells) were transfected with 12 μg of pCAGGS-SARS-CoV-2 Spike D614G (c-flag) plasmid using PolyFect transfection reagent (Figure S11), and 48 h later, incubated in 96-well flat bottom culture plates at a 1:1 ratio with 50,000 primary natural killer (NK) effector cells per well, in the presence of diluted plasma samples (1:100), together with interleukin-15 (IL-15, 40 ng/ml), brefeldin A (GolgiPlug, 1:1000), monensin (GolgiStop, 1:1500), and CD107a-FITC (clone H4A3), for 6 h at 37°C in a humidified 5% CO_2_ incubator. NK cells were then recovered into 96 well V-bottomed plates and stained for viability with Live/Dead Aqua fixable dead cell stain (Invitrogen), followed by a 20-min incubation with a monoclonal antibody cocktail consisting of CD56-PECy7 (clone NCAM16.2) and CD16-PacBlue (clone 3G8) at room temperature, in the dark. The NK cells were fixed with 1% paraformaldehyde and acquired on a Flow cytometer (BD FACS Melody), using the gating strategy in Figure S12.

To determine the percentage of live CD56^+^CD16^+^ CD107a+ NK cells as a measure of net spike-specific ADCC effector function, mock-assay activity in wells without SARS-CoV-2 spike expression was subtracted from wells with spike-transfected target cells. The CD107a gate was determined using NK cells incubated and stained in the absence of target HEK-293T/17 cells and plasma (antibodies). Target and effector cell wells (Spike+ HEK-293T/17 and NK cells only) were also included to subtract any background degranulation occurring in the absence of antibodies (i.e., baseline degranulation of the cells), Figure S13. Assay controls included a Phorbol 12-myristate 13-acetate and ionomycin (PMA/Ionomycin) cell activation cocktail to check for maximal degranulation potential of the NK cells, a CD16 monoclonal antibody and serum positive control, as well as one negative control; the CR3022 anti-Spike IgG1 monoclonal antibody (Figure S14A). NK cell degranulation/CD107a expression concomitant with downregulation of CD16 (FcγIIIa) receptors were correlated (Figure S14B). The CD16 down regulation index for each sample was obtained by computing the ratio of the CD16 median fluorescence intensity (MFI) of the target + effector cells only wells (i.e., wells with only Spike+ HEK-293T/17 and NK cells, without antibody) to that of the assay sample wells (i.e., wells with antibody, Spike+ HEK-293T/17 and NK cells). The net ratio was obtained by factoring off the non-spike specific downregulation in mock-assay wells in which untransfected HEK-293T/17 cells were used. We also showed correlation between percentage CD107a expression and CD107a MFI (Figure S14C).

### Quantification and statistical analysis

Analyses were done in R software (v4.4.2, Pile of Leaves) and FlowJo (v10.10.0). Inter-group differences in antibody responses were evaluated by pairwise Wilcoxon tests for matched data points and unpaired Mann Whitney U tests wherever there were unmatched data points, all with Benjamini-Hochberg positive false discovery rate (pFDR) corrections for multiple testing. Spearman’s rank correlation tests were used to evaluate relationships between antibody binding, neutralisation and ADCC profiles among groups of participants. In all tests, levels of statistical significance were determined at a threshold of *p* ≤ 0.05, with *p* > 0.05 being deemed not significant (ns). Significance levels were denoted as; ∗ for *p* ≤ 0.05, ∗∗ for *p* < 0.01, ∗∗∗ for *p* < 0.001, and ∗∗∗∗ for *p* < 0.0001.
