# Supplementary material for: Neutralization and ADCC reveal divergent spike-subdomain targeting across SARS-CoV-2 vaccine platforms in an African cohort
Source: iScience. 2025 Dec 5;29(2):114351. doi: 10.1016/j.isci.2025.114351 (PMC12857419; doi:10.1016/j.isci.2025.114351)
Supplement: Document S1. Figures S1–S14 and Table S1 [file mmc1.pdf]

iScience, Volume *29*

Supplemental information

Neutralization and ADCC reveal divergent spike-subdomain targeting across SARS-CoV-2 vaccine platforms in an African cohort

Gerald Kevin Oluka, Joseph Ssebwana Katende, Laban Kato, Violet Ankunda, Jackson Sembera, Peter Ejou, Geoffrey Odoch, Angella Namuyanja, Pontiano Kaleebu, and Jennifer Serwanga

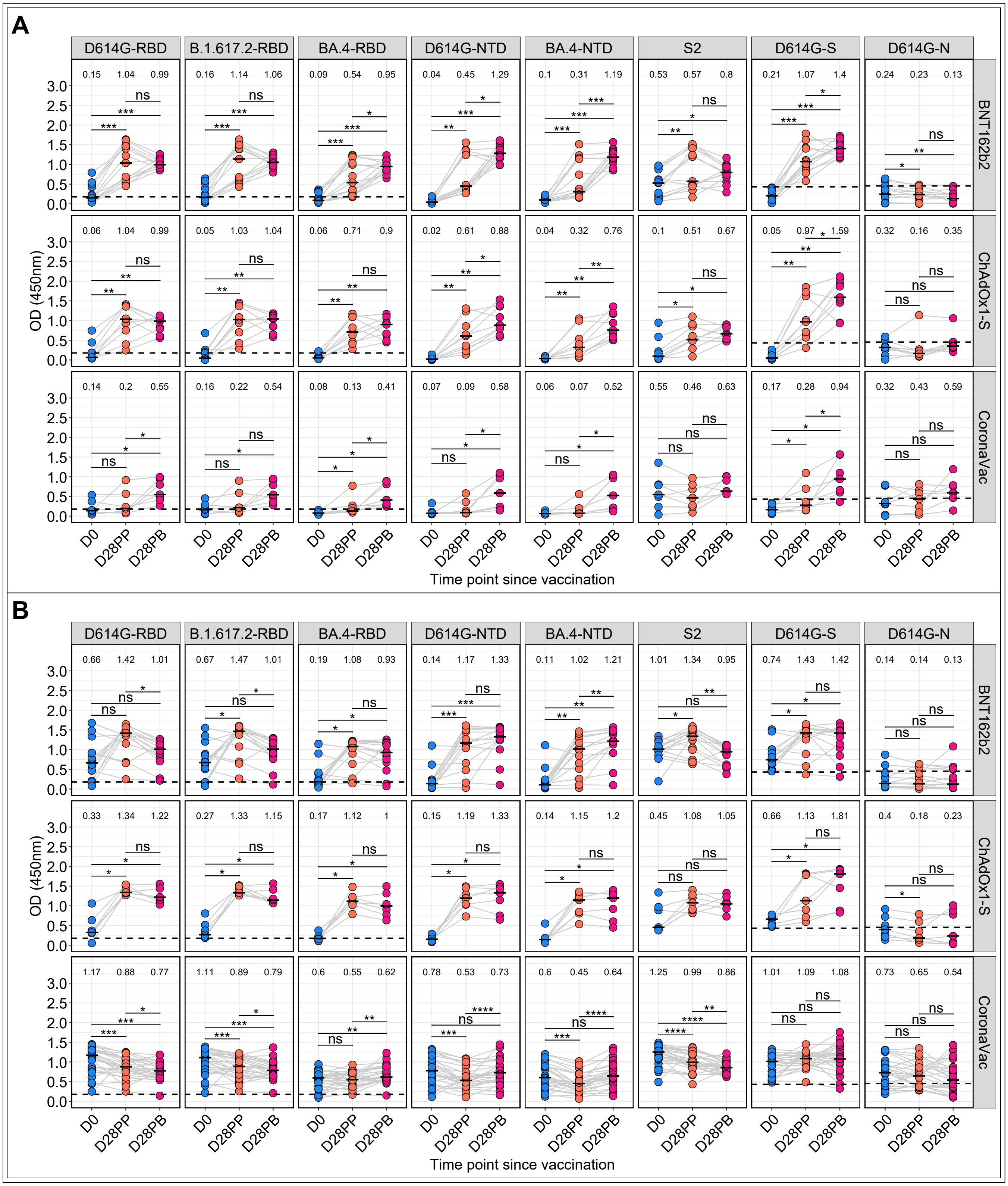

Figure S1. Longitudinal profiles of RBD, NTD, S2 spike-IgG binding antibody responses

Optical density (OD) values at D0 (baseline), 28 days post-primary vaccination (D28PP) and 28 days post-booster vaccination (D28PB) are shown for participants who were baseline S-IgG seronegative **(A)** and seropositive **(B)**. Crossbars represent median OD values at each time point. Dotted horizontal lines indicate seropositivity thresholds: 0.178 for RBD-IgG, 0.432 for S-IG and 0.454 for N-IgG. Statistical comparisons between median responses at different time points were performed using paired Wilcoxon tests with Benjamini-Hochberg correction for multiple testing. Statistical significance is indicated as ns (p-values > 0.05, not significant), * ($p \leq 0.05$), ** ($p < 0.01$), *** ($p < 0.001$) and **** ($p < 0.0001$).

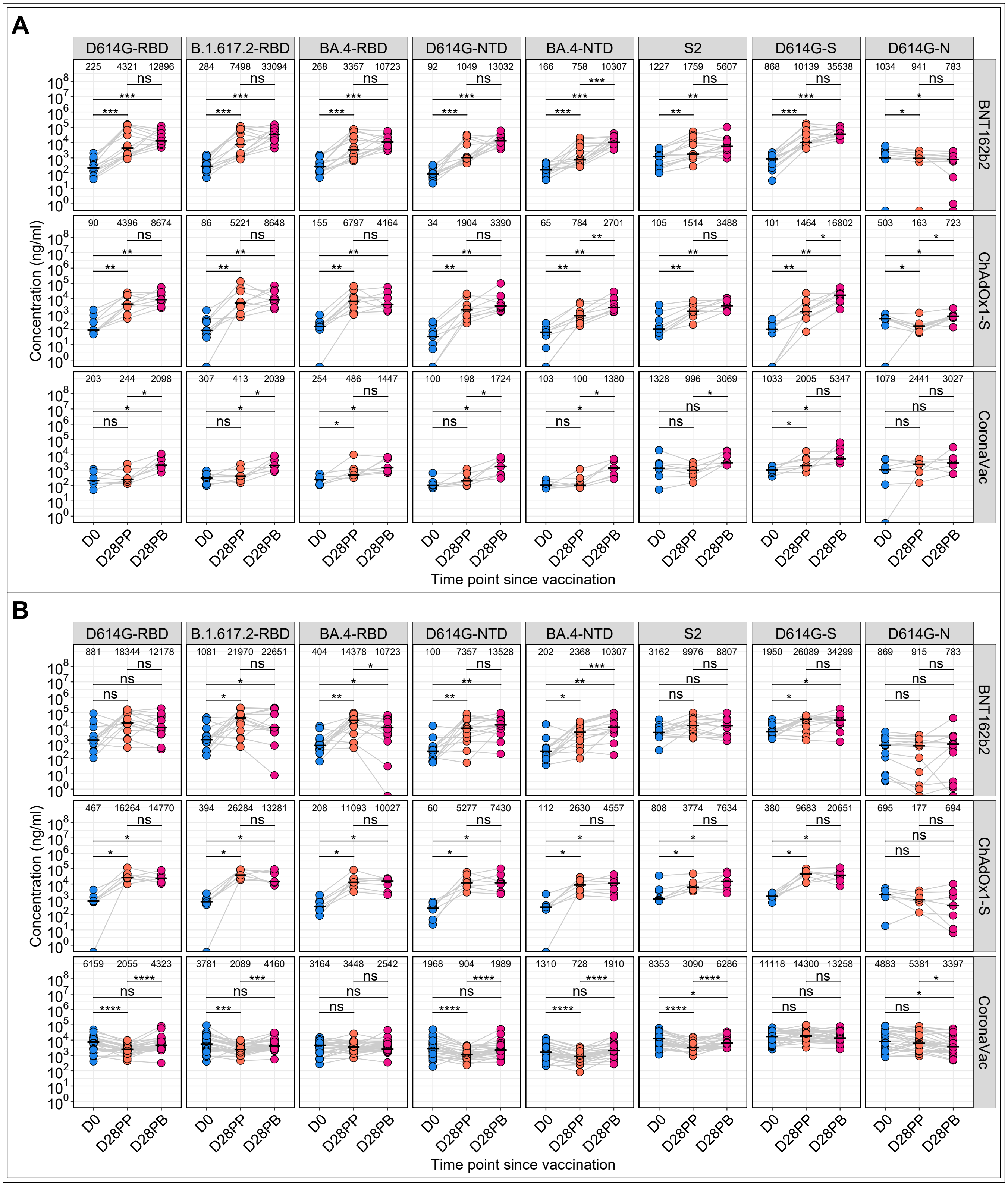

Figure S2. Longitudinal profiles of RBD, NTD, S2 spike-IgG binding concentrations

Concentrations (ng/ml) measured at D0, D28PP and D28PB are shown for baseline S-IgG- **(A)** and baseline S-IgG+ **(B)** participants. Cross-bars in data points represent median responses at each time-point. Significant differences between median responses at different time points were evaluated by paired Wilcoxon tests with Benjamini-Hochberg correction for multiple testing. Statistical significance is denoted by; ns (p-values > 0.05, not significant), * ($p \leq 0.05$), ** ($p < 0.01$), *** ($p < 0.001$) and **** ($p < 0.0001$).

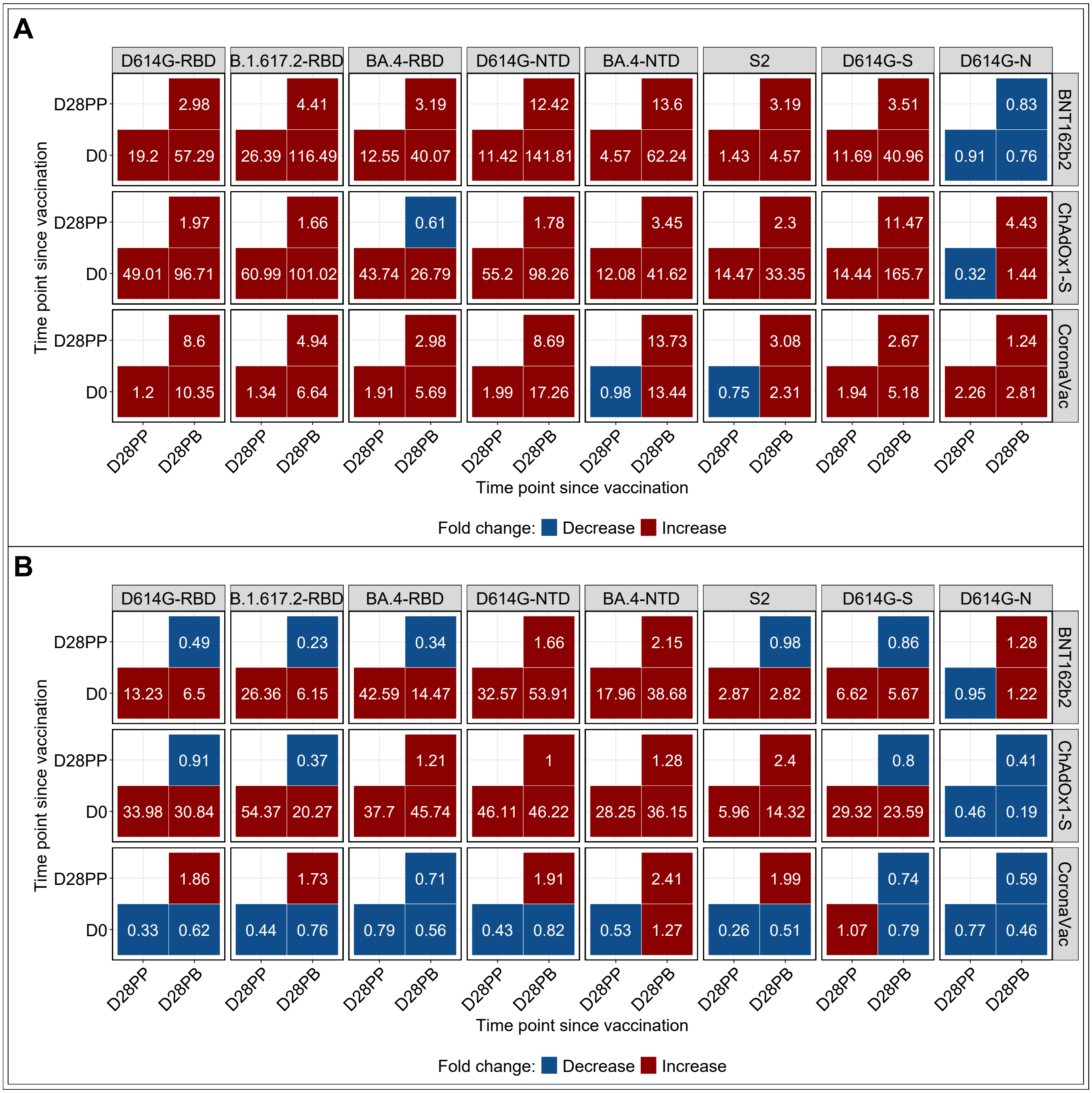

Figure S3. Longitudinal fold changes in RBD, NTD, S2-IgG concentrations

Pair-wise fold-changes in IgG antibody concentrations are shown across the three time points; D0, D28PP and D28PB, for participants who were baseline S-IgG- **(A)** and baseline S-IgG+ **(B)**. Blue colors represent fold-decrease while red colors represent fold-increase.

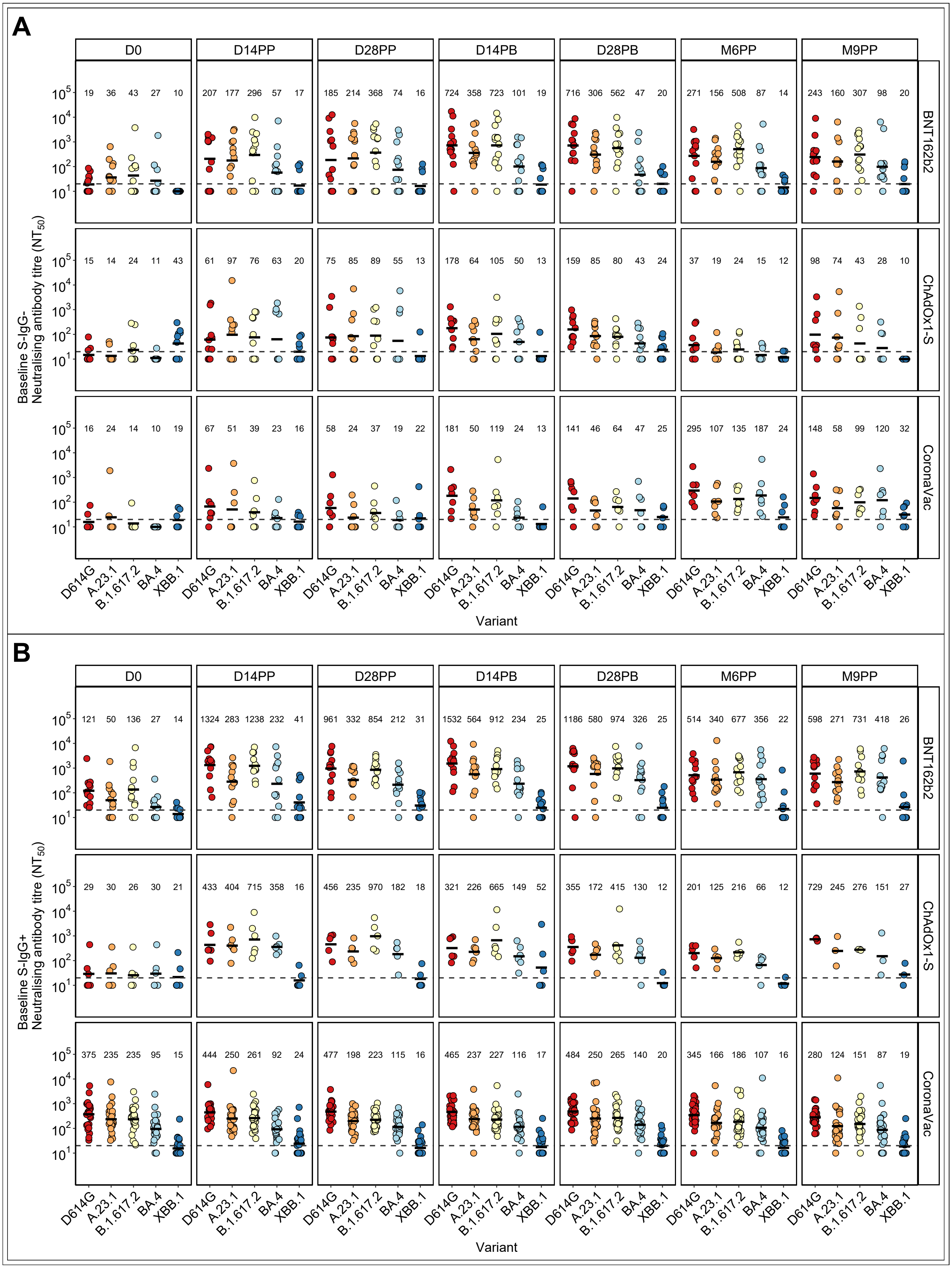

Figure S4. Longitudinal comparisons of inter-variant neutralising antibody responses

Comparisons are shown for variant-specific Geometric Mean Titres (GMT) in baseline S-IgG- **(A)** and baseline S-IgG+ participants **(B)** across longitudinal time points from D0 to M9PP. Dashed horizontal lines indicate limit of detection (NT_{50} = 20); values below this were assigned NT_{50} = 10.

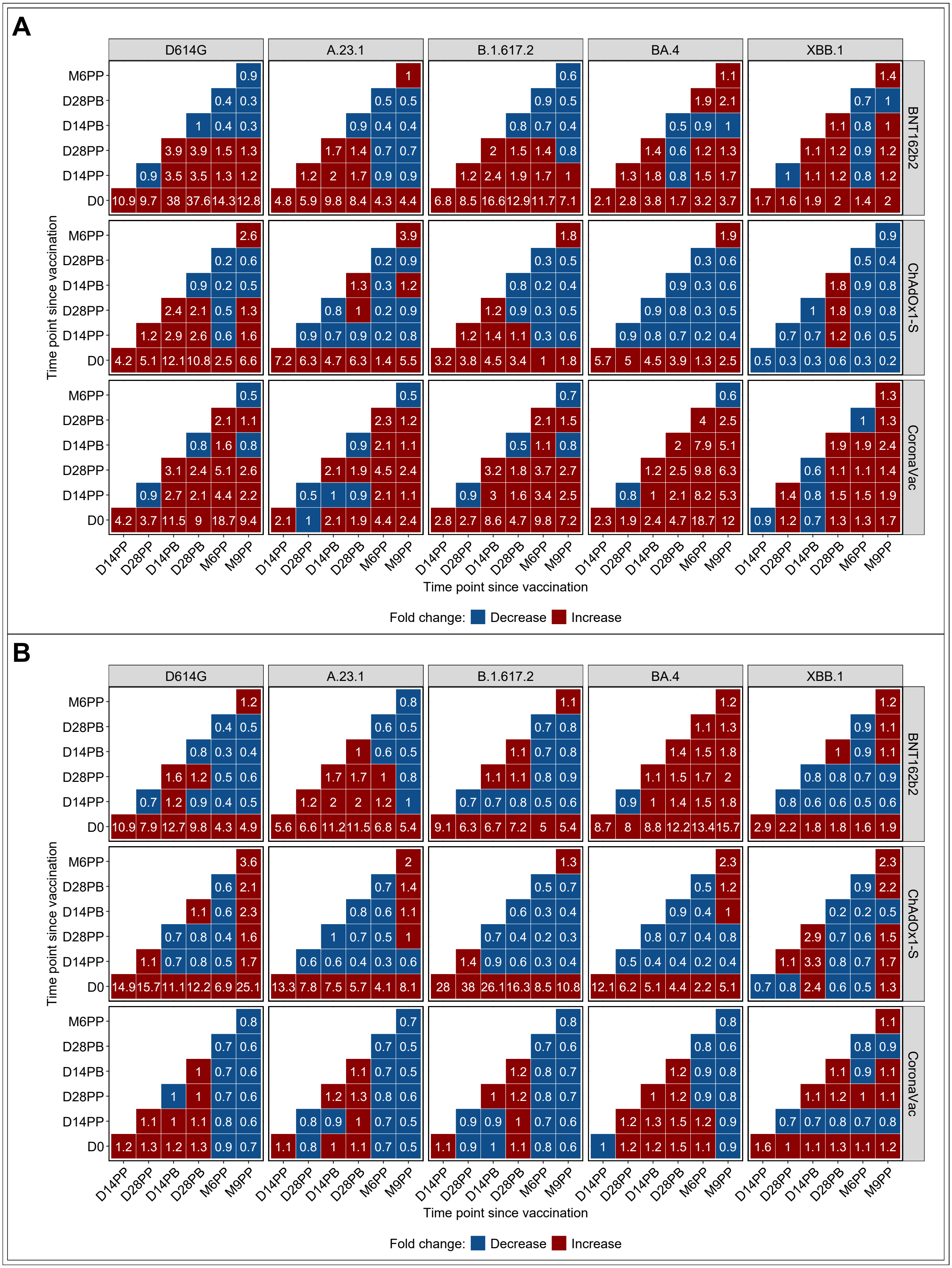

Figure S5. Longitudinal Fold-changes in Neutralisation Geometric Mean Titres

Pairwise fold-changes in Geometric Mean Titres (GMT) from one time point to another are shown for baseline S-IgG- **(A)** and baseline S-IgG+ participants **(B)**. Blue colors represent fold-decrease while red colors represent fold-increase.

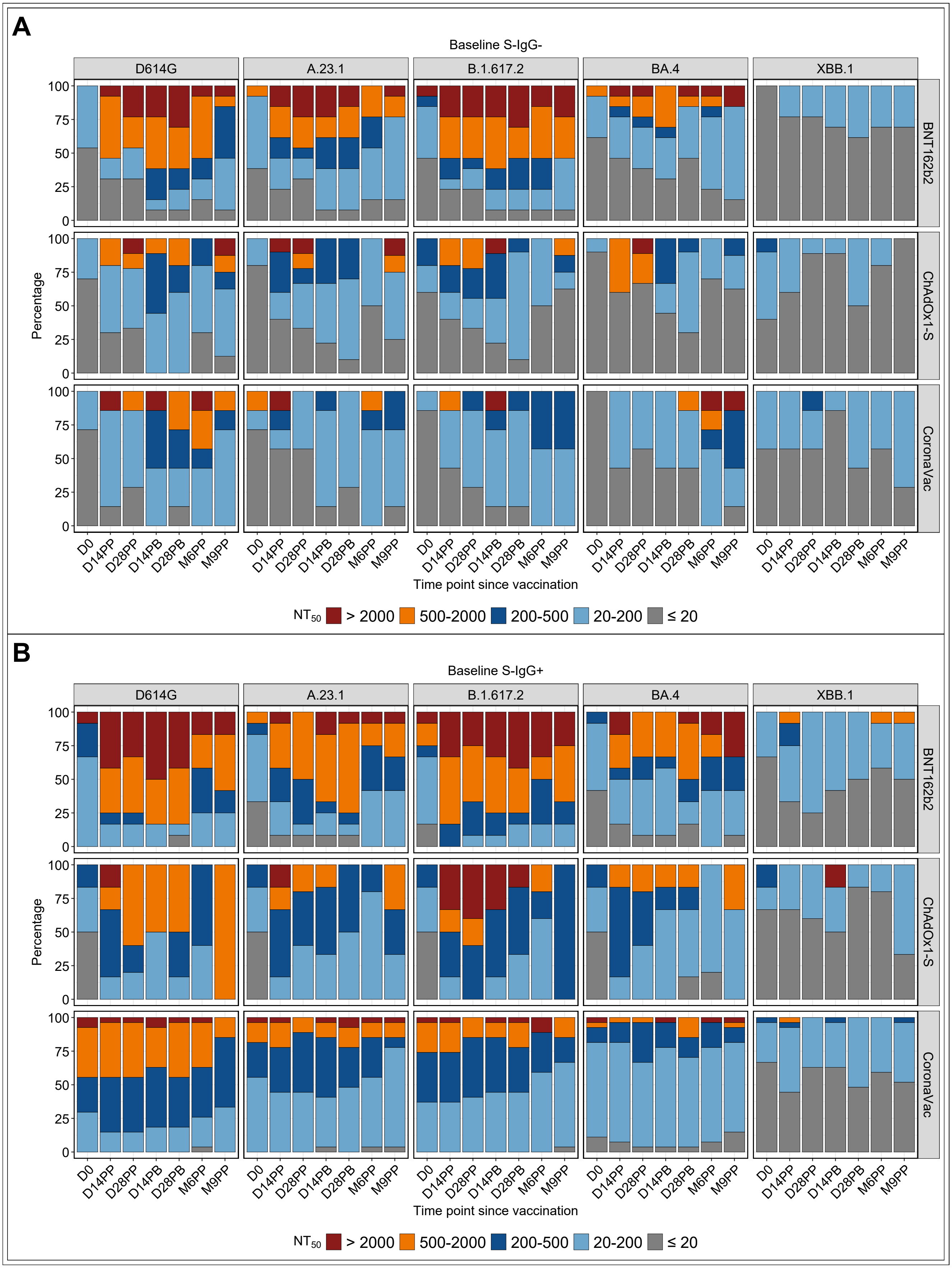

Figure S6. Potency of neutralising antibody responses

Percentages of baseline S-IgG- **(A)** and S-IgG+ **(B)** participants exhibiting undetectable ($NT_{50} < 20$), low (NT_{50} = 20–200), medium (NT_{50} = 200–500), high (NT_{50} = 500–2,000) or potent/extremely high ($NT_{50} > 2,000$) neutralising antibody titres against each SARS-CoV-2 variant over time.

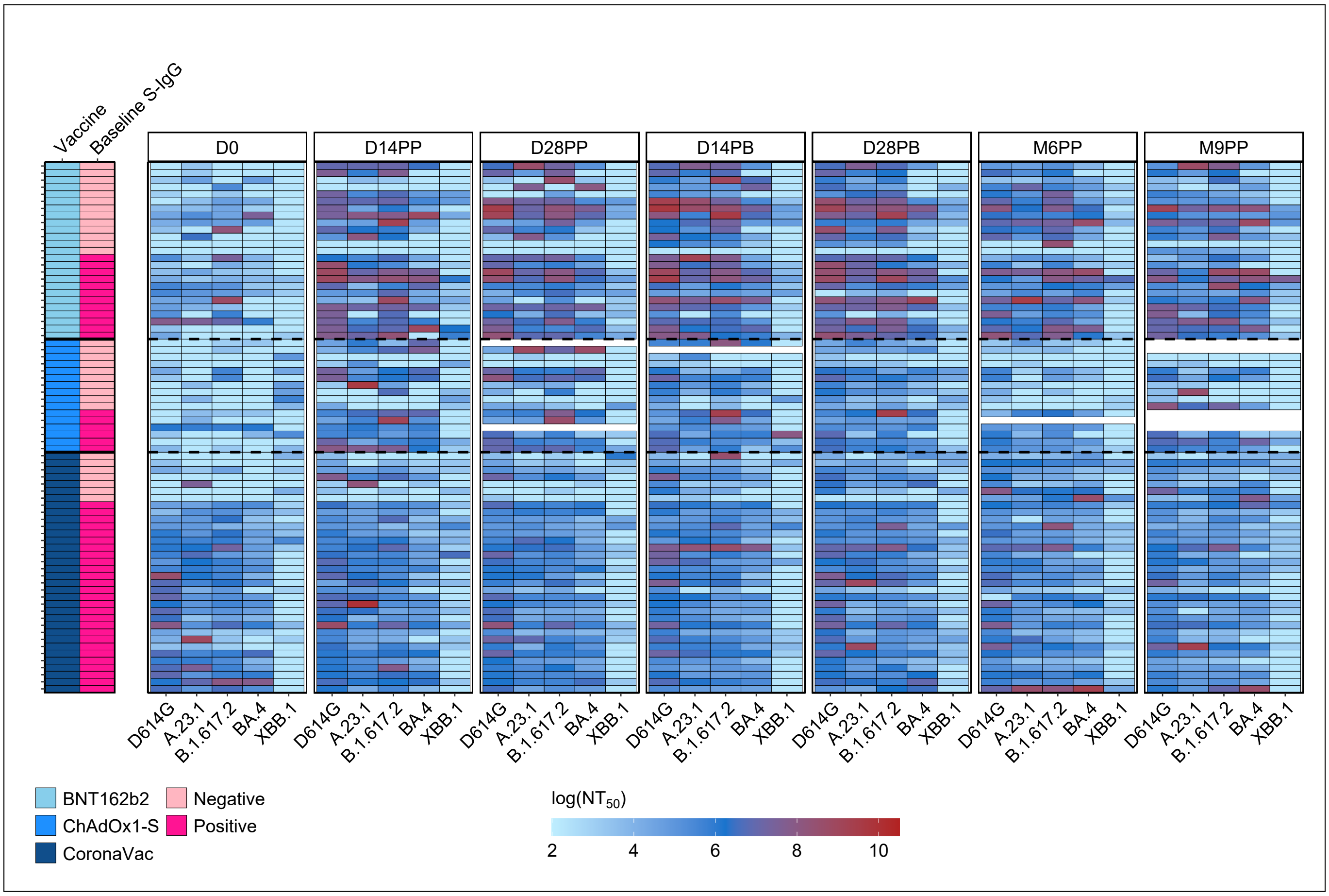

Figure S7. Magnitude and breadth of neutralising antibody responses over time

Participant-specific response profiles are shown as horizontal lines across five variants of SARS-CoV-2, starting at D0 to M9PP. The color gradient from light blue to dark red indicates the magnitude of responses.

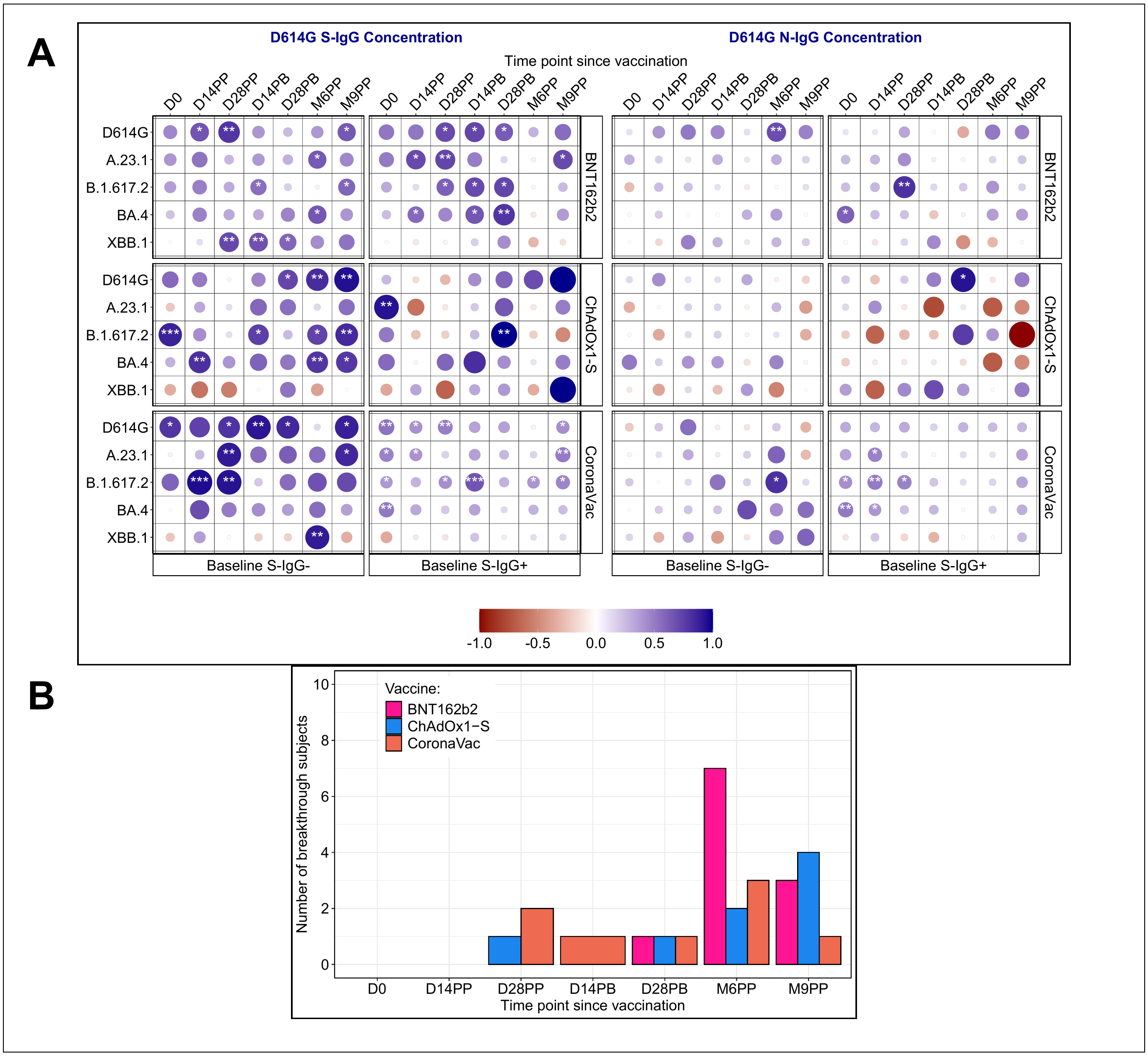

Figure S8. Correlations between neutralising antibody titres, S-IgG and N-IgG binding antibody concentrations and longitudinal tracking of breakthrough infections

(A) Plots of Spearman's rank correlations between neutralising antibody titres (NT_{50}) and spike-IgG or nucleoprotein-IgG antibody concentrations (ng/ml) are shown at each time point. The size of the circles shows relative strength of correlation, with bigger circles showing stronger correlations. The blue color gradient shows positive correlations while the red color gradient shows negative correlations. Statistical significance was considered at $p \leq 0.05$, and denoted as; ns (p-values > 0.05, not significant), * ($p \leq 0.05$), ** ($p < 0.01$), *** ($p < 0.001$) and **** ($p < 0.0001$). For visual clarity, only statistically significant correlations are indicated. **(B)** Longitudinal tracking of numbers of participants with breakthrough infections at each time point for each vaccine group, determined by a $\geq$ 11-fold increase in N-IgG antibody concentration.

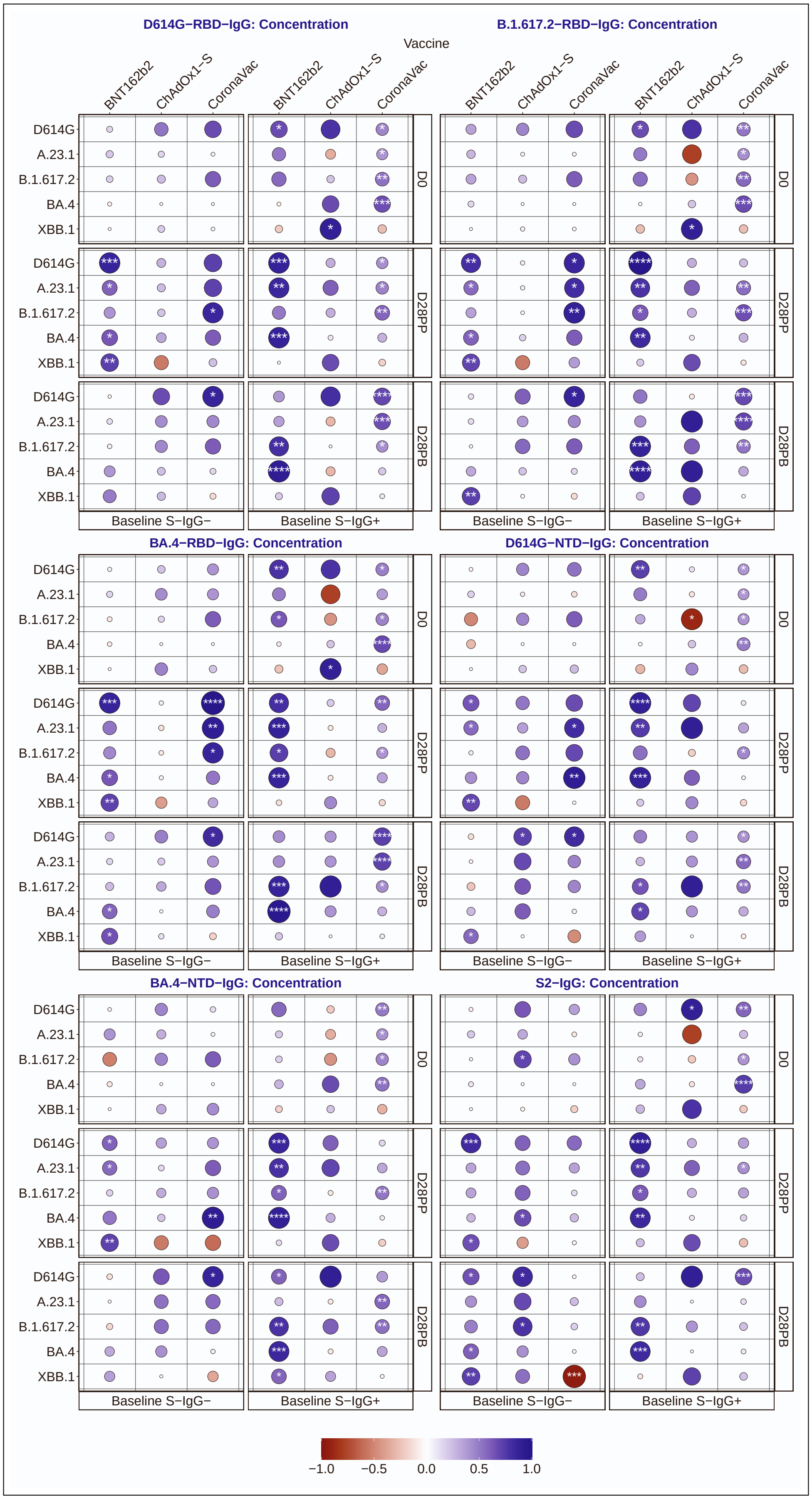

Figure S9. Correlations between neutralising antibody titres and RBD, NTD and S2-IgG concentrations
Plots of Spearman's rank correlations between neutralising antibody titres (NT_{50}) and spike-IgG or nucleoprotein-IgG antibody concentrations (ng/ml) are shown at each time point. The size of the circles shows relative strength

of correlation, with bigger circles showing stronger correlations. The blue color gradient shows positive correlations while the red color gradient shows negative correlations. Statistical significance was considered at $p \leq 0.05$, and denoted as; ns (p-values > 0.05, not significant), * ($p \leq 0.05$), ** ($p < 0.01$), *** ($p < 0.001$) and **** ($p < 0.0001$). For visual clarity, only statistically significant correlations are indicated.

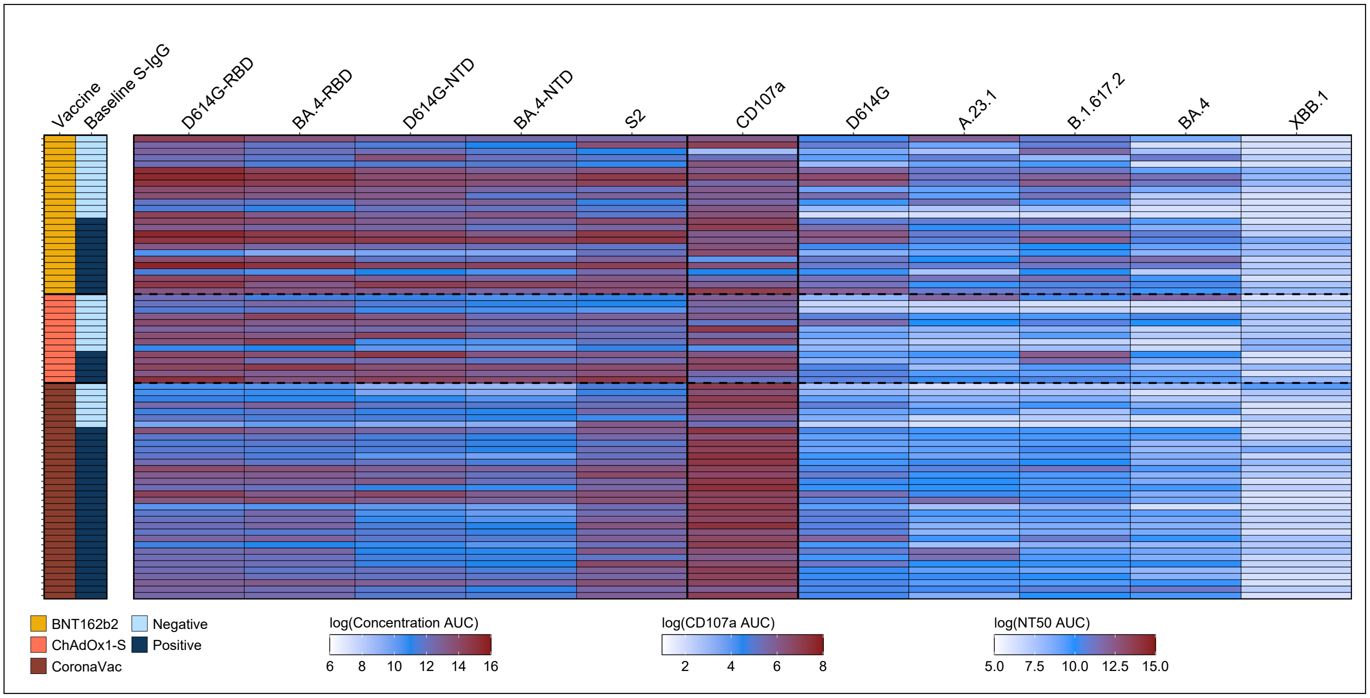

Figure S10. Comparison of RBD, NTD and S2-IgG binding, ADCC effector function, and neutralisation
Participant-specific response profiles presented as time-dependent areas under the curve (AUC) for spike-(RBD, NTD, S2)-IgG binding concentrations, ADCC as percentage expression of CD107a, and neutralising antibody titres (NT_{50}) for each variant, are visualized in color gradients ranging from light blue to dark red.

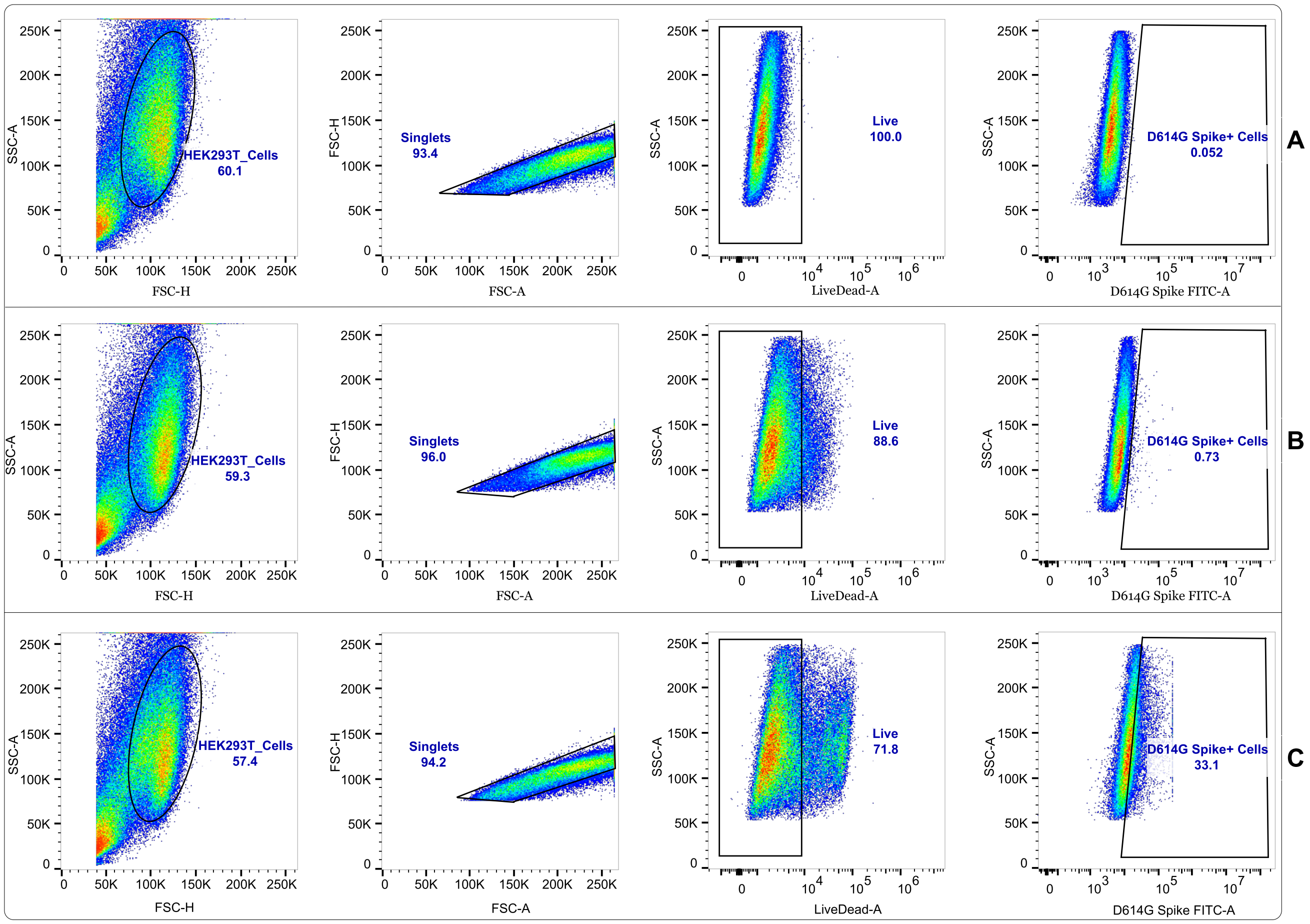

Figure S11. Cell-surface expression of D614G spike protein on HEK293T/17 cells

As a surrogate for SARS-CoV-2 infected cells, HEK-293T/17 mammalian cells transiently transfected with D614G spike were evaluated for surface expression and are shown in their normal healthy state without transfection and staining with antibody-fluorophore complex **A)**, without transfection, but fully stained, to assess background levels of non-specific expression **(B)**, and finally, after spike protein transfection and anti-spike-tag antibody-fluorophore/FITC complex staining **(C)**. The spike-tag was a FLAG-octapeptide (DYKDDDDK).

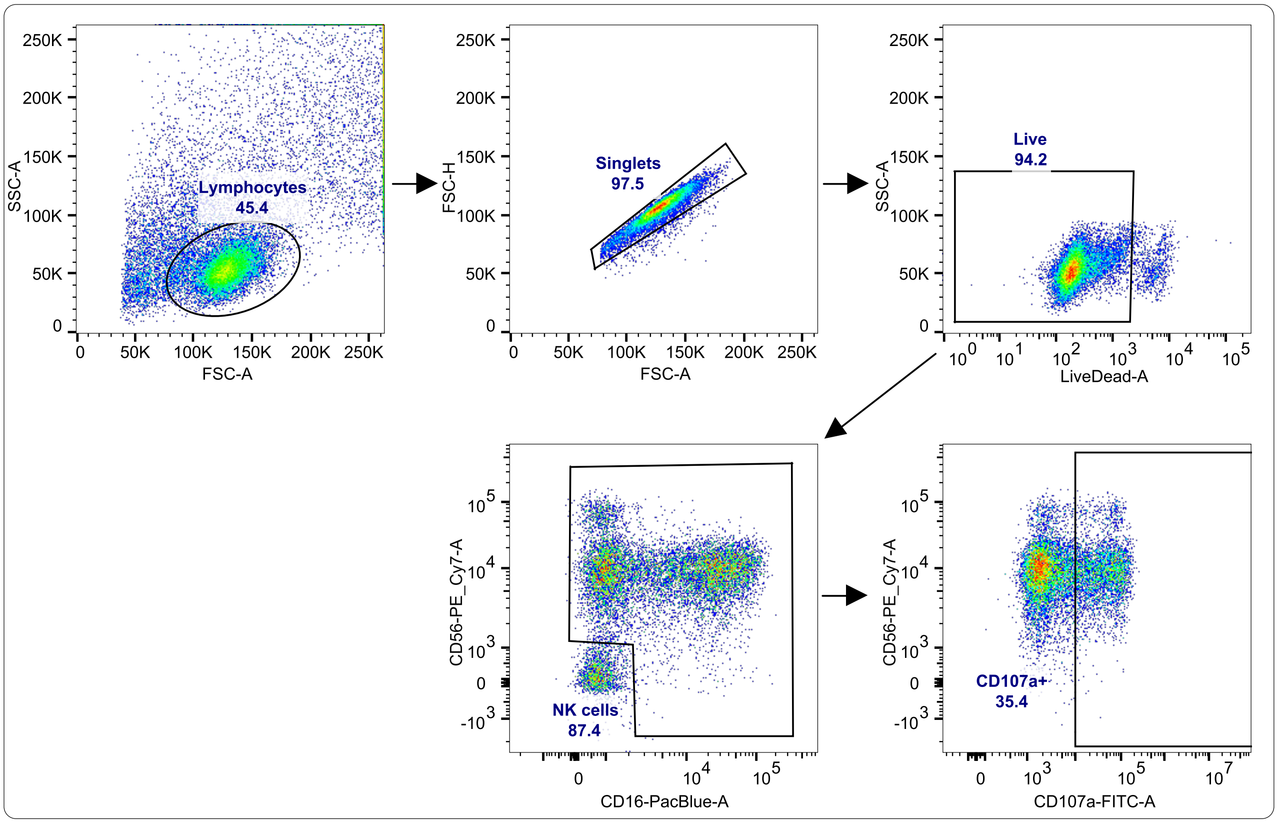

Figure S12. Gating strategy used in the NK cell degranulation assay (ADCC)

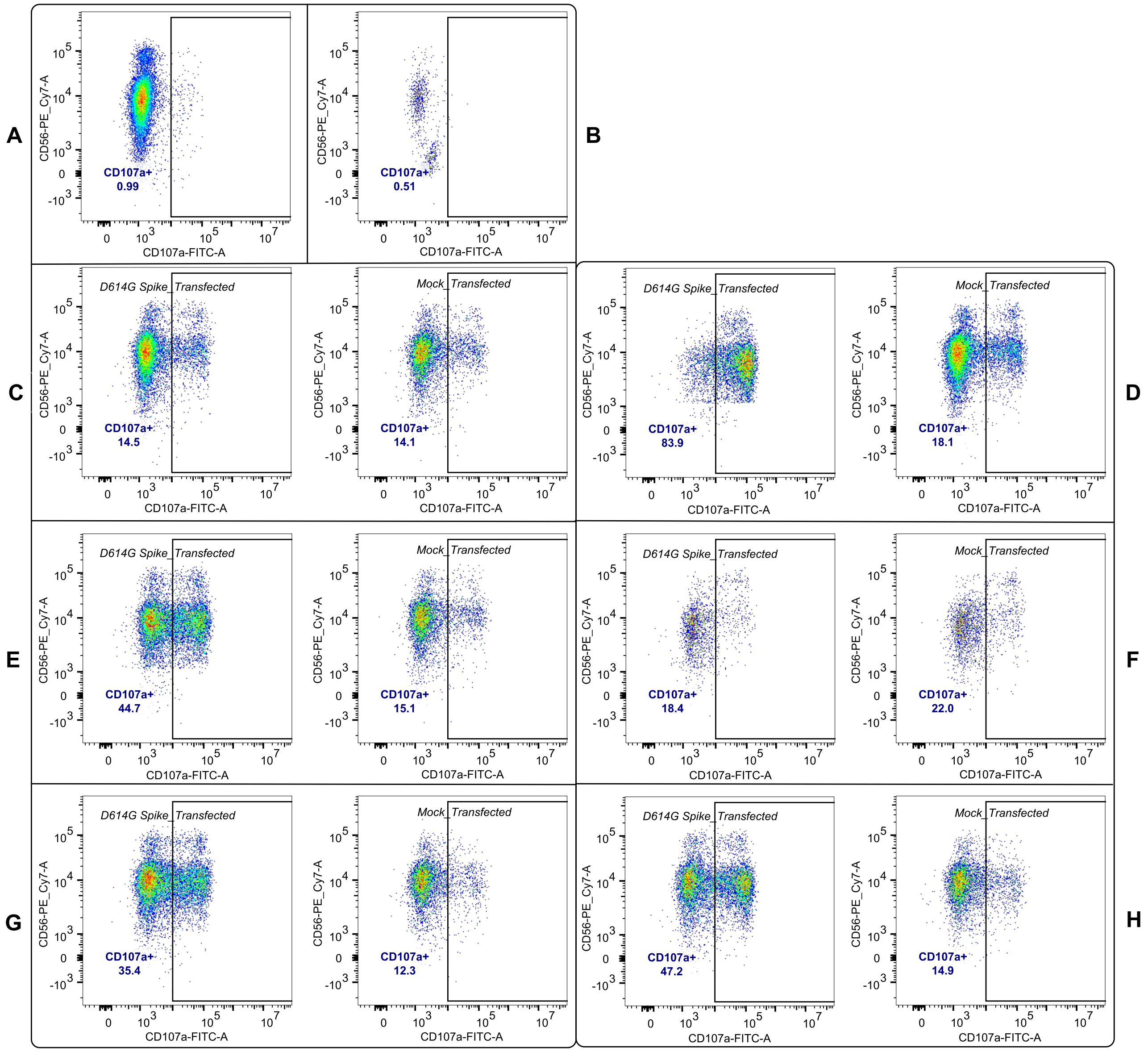

Figure S13. CD107a gates for assay (transfected) and mock (untransfected) controls Gates of percentage CD107a expression are shown for unstained primary NK cells **(A)**, fully stained primary NK cells **(B)**, NK cells and Spike+ HEK-293T/17 cells only **(C)**, PMA/Ionomycin control — added to only the left panel **(D)**, anti-CD16 mAb — added to only the left panel **(E)**, CR3022 mAb **(F)**, serum positive control **(G)** and one participant's assay sample **(H)**. mAb: monoclonal antibody. PMA: Phorbol 12-myristate 13-acetate

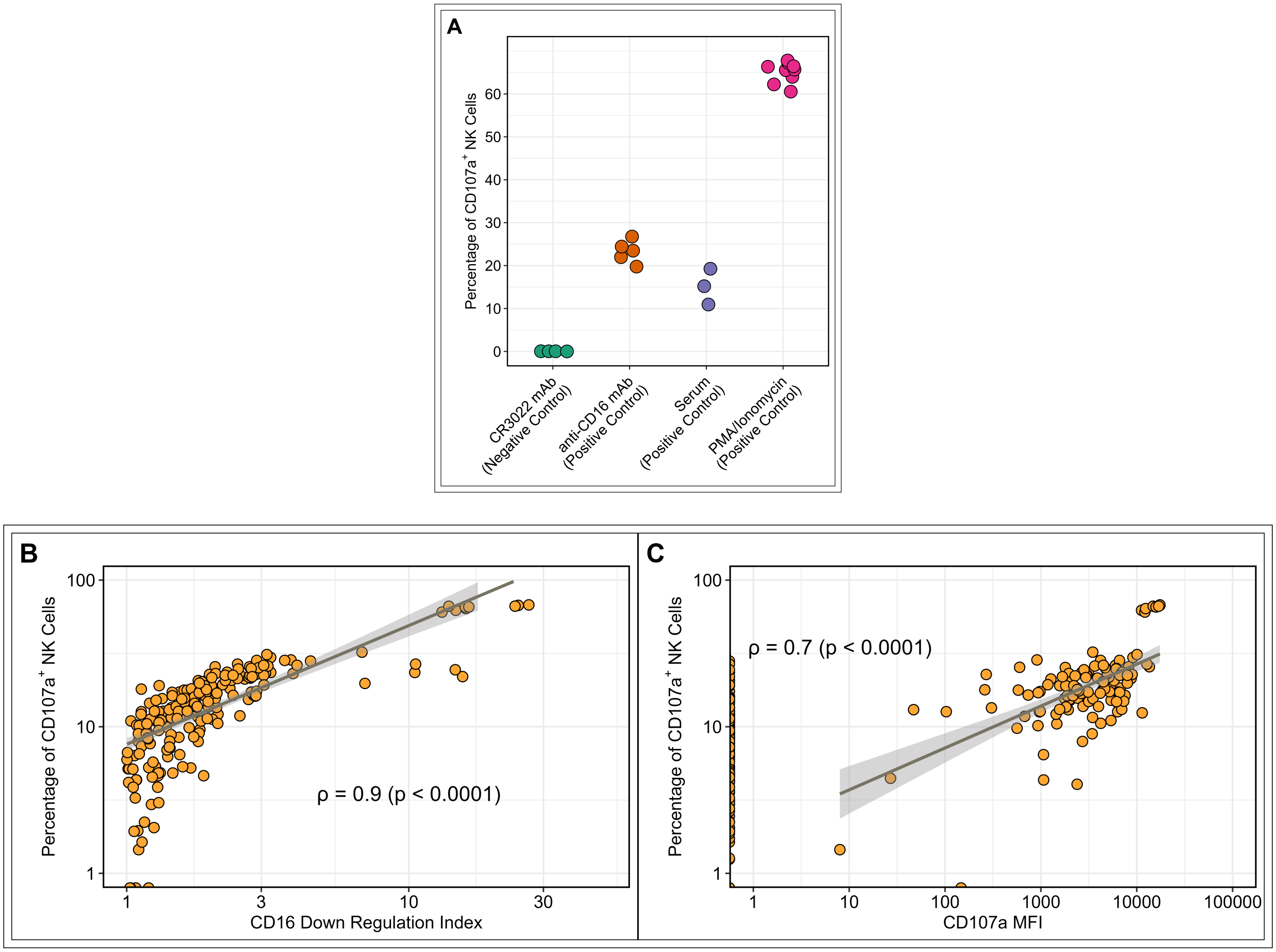

Figure S14. Assay control performance and correlations between percentage CD107a expression and CD16 down regulation, and CD107a MFI

(A) Percentage CD107a expression of negative and positive controls in the NK cell degranulation assay. Spearman's rank correlation tests were done between percentage CD107a expression and CD16 (FcγRIIIa) downregulation **(B)**, as well as CD107a median fluorescence intensity (MFI) **(C)**. Statistical significance was considered at $p \leq 0.05$.

Table S1. Summary of participant age distribution

Vaccine	Participants n (%)	Age-Range (Years)	Median Age (IQR)
Total	80 (100%)	18-56	27 (21.0-33.5)
BNT162b2	27 (34%)	19-49	30 (27.0-36.0)
ChAdOx1-S	18 (23%)	20-56	29 (26.0-40.0)
CoronaVac	35 (44%)	18-41	21 (20-25.5)
